# GSK3β inhibits the differentiation of follicular granulosa cells by promoting lipid accumulation through autophagy in chickens

**DOI:** 10.1016/j.psj.2026.107399

**Published:** 2026-07-05

**Authors:** Yuechen Liao, Ashi Li, Yangqiwen Luo, Cangning Zhang, Meng Ma, Genxi Zhang, Liumei Sun, Jiying Liu, Manman Shen, Liang Qu

**Affiliations:** a Jiangsu Key Laboratory of Sericultural and Animal Biotechnology, School of Biotechnology, Jiangsu University of Science and Technology, Zhenjiang, 212100, China; b Key Laboratory of Silkworm and Mulberry Genetic Improvement, Ministry of Agriculture and Rural Affairs, Sericultural Scientific Research Center, Chinese Academy of Agricultural Sciences, Zhenjiang, 212100, China; c Jiangsu Institute of Poultry Science, Yangzhou, 225125, China; d Jiangsu Key Laboratory of Animal Genetic Breeding and Molecular Design, College of Animal Science and Technology, Yangzhou University, Yangzhou, 225009, China

**Keywords:** Glycogen synthase kinase 3 beta, Autophagy, Lipid metabolism, Mitochondria, Granulosa cells

## Abstract

Granulosa cells (GCs) are integral to the process of follicular development in poultry, with their differentiation and hormone synthesis being critical for follicle selection. While glycogen synthase kinase 3 beta (GSK3β) is recognized as a significant regulator of energy metabolism, differentiation, and autophagy, its specific function within GCs remains unclear. Elucidating the role of GSK3β in GCs is essential for deciphering the mechanisms governing follicle selection. Our in vitro studies in GCs from prehierarchical follicles demonstrated that overexpression of GSK3β inhibited both differentiation and proliferation, while simultaneously promoting steroid hormone synthesis. Conversely, GSK3β knockdown yielded the opposite effects. Transcriptomic analyses, supplemented by further validation, revealed that overexpression of GSK3β initiated autophagy and lipid metabolism but impeded autophagic flux, as evidenced by increased LC3-II levels and elevated p62 accumulation. Furthermore, GSK3β overexpression resulted in enhanced intracellular lipid droplet accumulation. These effects, along with the observed rise in progesterone levels and reduction in FSHR levels, were attenuated by co-treatment with rapamycin (Rapa). Mechanistically, our findings suggest that the impairment of autophagic flux induced by GSK3β triggers lipid accumulation, leading to elevated mitochondrial damage and lipid peroxidation, which in turn negatively affects GC differentiation. In conclusion, GSK3β disrupts follicular GC function by initiating autophagy while blocking its flux. This disruption induces excessive lipid accumulation, ultimately inhibiting GC differentiation. This study provides novel insights into the role of GSK3β in poultry follicular development and offers a new theoretical framework for understanding the mechanisms of follicle selection.

## Introduction

Egg production is a critical determinant of economic efficiency in the poultry industry. Ovarian function and follicular development are intricately linked to egg production outcomes. In poultry, follicular development is a complex and dynamic process involving initial and cyclical recruitment, during which follicles progress through a series of distinct developmental stages (Johnson, 2015; Li et al., 2022b). During the laying period, follicles that successfully undergo cyclical recruitment and subsequent selection advance to the hierarchical follicle stage, ultimately leading to ovulation and completion of the egg-laying process (Johnson, 2012). Conversely, pre-hierarchical follicles that do not transition to the hierarchical follicle stage undergo atresia and degeneration (Johnson, 2015). The orderly progression of follicle selection is essential for maintaining consistent laying performance (Liu et al., 2022). Therefore, facilitating effective follicular selection is a crucial strategy for ensuring the orderly development of hierarchical follicles. Nevertheless, follicular development and the follicle selection process are influenced by multiple factors, including reproductive hormones, genetics, and transcriptional regulation (Du et al., 2020; Hernandez and Bahr, 2003; Nie et al., 2024; Zhao et al., 2023).

The follicle primarily consists of an oocyte, a granulosa cell (GC) layer, and a theca cell layer (Zhang et al., 2025). During follicle selection, the differentiation, hormone synthesis, proliferation, and apoptosis of GCs play crucial roles in determining whether a follicle progresses to the dominant or hierarchical stage (Johnson, 2015). During this process, follicle-stimulating hormone receptor (FSHR) expression levels in GCs significantly increase, enabling a rapid response to FSH (Johnson and Woods, 2009) and inducing substantial changes in cellular morphology, structure, and function (Johnson, 2015; Kim et al., 2013). Differentiation of GCs is primarily characterized by the acquisition of specialized responsiveness to FSH once a single follicle is selected into the hierarchy stage (Johnson, 2015; Johnson, 2012). Following GC differentiation, alterations in FSHR expression influence intracellular gene expression and signaling pathways. Elevated FSHR expression amplifies cAMP signaling cascades, promoting the transcription and expression of genes associated with cell proliferation and hormone synthesis, thereby facilitating rapid GC proliferation and enhancing their steroidogenic capacity (Zhang et al., 2024a), resulting in hierarchical follicles. In contrast, GCs in unselected follicles undergo apoptosis, ultimately leading to follicular atresia (Johnson, 2015). In the selected follicle, these processes of proliferation, differentiation, and steroidogenesis operate in a highly synchronized and balanced manner. However, any disruption of homeostasis that uncouples GC proliferation from appropriate differentiation can precipitate steroidogenic dysregulation or premature differentiation-arrest phenotypes (Wang et al., 2017). Therefore, investigating factors that influence GC differentiation is a central research focus for elucidating the mechanisms underlying follicular development.

Autophagy, an evolutionarily conserved intracellular degradation mechanism, is essential for maintaining cellular homeostasis (Mizushima and Komatsu, 2011). In the ovaries, autophagy is active during oocyte formation, follicular development, and regression (Kumariya et al., 2021). It plays a complex and critical role in GCs, where the accumulation of autophagosomes triggers apoptosis, thereby facilitating follicular atresia (Choi et al., 2014). Recent investigations in avian follicular GCs have demonstrated that autophagy inhibition leads to a reduction in primordial follicles and delays in follicular development (Dong et al., 2022b), whereas enhanced autophagy delays follicular aging (Bai et al., 2024), reduces apoptosis (Jiang et al., 2023), and inhibits follicular atresia (Dong et al., 2022a). Importantly, emerging evidence indicates that autophagy is tightly linked to lipid metabolism during follicular development. In mammalian systems, autophagy regulates lipid homeostasis and steroidogenesis by mediating the lysosomal degradation of lipid droplets, thereby influencing the availability of cholesterol substrates required for steroid hormone synthesis in ovarian and testicular tissues (Yamada et al., 2025). Dysregulation of autophagy-mediated lipid metabolism has therefore been implicated in impaired follicular development and reproductive dysfunction (Bassi et al., 2021). However, how autophagy regulates lipid metabolism in poultry GCs has been rarely investigated.

Glycogen synthase kinase 3 beta (GSK3β) encodes a serine/threonine protein kinase that serves as a key regulator in multiple intracellular signaling pathways. It participates in various biological processes, including energy metabolism, cell differentiation, and stress response regulation (Beurel et al., 2015). As a critical regulatory component of the Wnt signaling pathway, GSK3β is involved in follicular development (Habara et al., 2021). Inhibition of GSK3β enhances Wnt signaling, thereby facilitating osteoblast differentiation (Galli et al., 2013). Furthermore, GSK3β serves as an upstream regulator of autophagy by inducing this process through the phosphorylation of ULK1 in rat neuronal stem cells (Ryu et al., 2021). Recent studies in poultry have demonstrated that FSH stimulation significantly increases the expression of phosphorylated GSK3β in chicken follicular GCs, which contributes to the attenuation of GC senescence (Dong et al., 2022a), while FSH significantly decreased GSK3β mRNA expression levels in vitro cultured GCs (Shen et al., 2022). A study by Zhang et al. (2024b) also revealed that GSK3β modulates cholesterol uptake in chicken theca cells, thereby affecting estrogen synthesis. Nonetheless, the specific regulatory mechanisms of GSK3β in chicken GC function remain to be elucidated. Therefore, this study aims to investigate the regulatory role and molecular mechanisms of GSK3β in the differentiation, proliferation, and hormone synthesis of GCs from prehierarchical follicles in chickens, providing a scientific basis for understanding follicular development and improving egg production performance in poultry.

## Materials and methods

### Granulosa cell culture

Prehierarchical follicles measuring 4 to 8 mm in diameter were collected from the ovaries and placed in PBS. Subsequently, GCs were isolated and cultured following the methodology described in our previously published work (Shen et al., 2023). Briefly, the yolk was meticulously removed from the follicles, and the GC layer was separated using ophthalmic techniques. The isolated GCs were washed three times with PBS and then cut into approximately 1 mm³ pieces. The cells were digested with 0.1% type II collagenase (Solarbio, Beijing, China) for 5 min at 37°C. Digestion was stopped by adding an equal volume of complete Dulbecco's Modified Eagle Medium (DMEM) supplemented with 10% fetal bovine serum (Biochannel, Nanjing, China), 100 U/mL penicillin, and 100 μg/mL streptomycin. The resulting cell suspension was filtered through a 70 μm sieve to obtain a single-cell suspension. Following centrifugation at 1000 rpm for 5 min, the cells were resuspended in complete DMEM and seeded into culture plates. The cells were incubated at 37°C in a 5% CO₂ environment. All chickens utilized in this study were obtained from the Jiangsu Institute of Poultry Science. Animal use was approved by the Ethical Committee of Jiangsu University of Science and Technology (Approval No. G2025SJ21). Our experiments were conducted with at least 3 independent biological replicates, each with three technical replicates. In total, about 50 hens were used in the current study.

### Cells transfection and treatment

For GSK3β overexpression, the full-length cDNA of GSK3β (XM_004938179) was amplified from ovarian tissue cDNA stored in our laboratory and inserted into the pcDNA-N-HA vector (EK-Bioscience, Shanghai, China). Small interfering RNAs (siRNAs) targeting GSK3β were designed and synthesized by GenePharma (Shanghai, China). For overexpression, pcDNA-N-HA-GSK3β (OE-GSK3β) and the vector plasmid (pcDNA 3.1) were transfected with Lipomaster 3000 (TL301, Vazyme, Nanjing, China), a reagent successfully applied for vehicle delivery and siRNA transfection in both mammalian and fish cells (Hu et al., 2025; Ke et al., 2026).

For RNA interference, GSK3β siRNAs and negative control siRNAs were transfected using Lipomaster 3000. The transfection protocol used in this study was adapted from these established methods (Hu et al., 2025; Ke et al., 2026), with specific procedures detailed as follows: transfection was performed when cultured cells reached 40–50% confluence, using either OE-GSK3β (2 μg per well in a 6-well plate) or si-GSK3β (final concentration of 50 nM). Six hours post-transfection, the medium was replaced with fresh complete DMEM. Cells and supernatants were harvested 48 h post-transfection for subsequent experiments. Primers for amplification and siRNAs targeting the GSK3β CDS are listed in Table 1.

**Table 1:** Amplification primers and siRNA sequences for GSK3β in the current study.

Primer name	Primer Sequence (5’-3’)	Accession No.
GSK3β-HA	F: GGAATTCatgtccgggcggcc R: CCGCTCGAGtcaggtggagttggaggctg	XM_416557.8
siRNA-149	S: GACCACAAGAAGUUAGCUATT AS: UAGCUAACUUCUUGUGUCTT	
siRNA-309	S: GCUGGAUCAUUGUAACAUUTT AS: AAUGUUACAAUGAUCCAGCTT	
siRNA-626	S: GUGGAGAACCUAACGUUUCTT AS: GAAACGUUAGGUUCUCCACTT	

Note: The underlined parts represent the added enzyme cleavage sites

To elucidate the role of GSK3β in autophagy regulation, cells were seeded and cultured until reaching approximately 40–50% confluence after seeding. The cells were then transfected with either an OE-GSK3β plasmid or GSK3β-specific siRNAs. After 24 hours of transfection, the cells were treated with fresh medium containing 1 μM rapamycin (Rapa, Selleck Chemicals, USA) according to a previous study (Shao et al., 2022) for an additional 24 h before sample collection for subsequent analyses.

### Cell viability assay

Cell viability of GCs was assessed using Cell Counting Kit-8 (CCK-8, K1018, APExBIO Technology LLC, USA). Briefly, GCs were seeded in 96-well plates at a density of 3.5 × 10^5^ cells/mL. Following GSK3β overexpression or knockdown, the cells were incubated with 10 μL of CCK-8 reagent per well at 37°C for 2 h. The OD at 450 nm was then measured using a microplate reader (Epoch 2, BioTek, USA). Cell viability was calculated according to the manufacturer's protocol.

### Measurement of progesterone

During the progesterone measurement assay, the culture medium was replaced with DMEM containing 0.5% FBS and 1% (v/v) insulin-transferrin-se (ITS, Sigma, Shanghai, China) solution (Shen et al., 2023) after the cells had undergone transfection and Rapa treatment. After treatment, culture supernatants were collected and stored at -20°C. Progesterone concentrations in the supernatants were measured using commercial ELISA kits (H089-1-1, Jiancheng, Nanjing, China) following the manufacturer's instructions.

### Immunofluorescence assay

After treatment, cells were fixed in 4% paraformaldehyde for 30 min at room temperature (20–25°C), permeabilized with 0.5% Triton X-100 for 20 min, and then blocked in 5% BSA at 37°C for 30 min. The cells were then incubated with an anti-FSHR primary antibody (22665-1-AP, Proteintech), followed by an anti-rabbit IgG secondary antibody (ab150077, Abcam). Nuclei were counterstained with DAPI. Images were captured using an IX73P1F microscope (Olympus, Tokyo, Japan) and analyzed with Fiji/ImageJ software (Schindelin et al., 2012).

### Western blot analysis

Total proteins were extracted from treated GCs using RIPA lysis buffer (P0013B, Beyotime, Shanghai, China) containing protease inhibitors (P1005, Beyotime). Protein concentrations were determined using the BCA Assay Kit (A045-4-2, Jiancheng). Proteins were mixed with 5× loading buffer (BL502, Biosharp, Hefei, China) and denatured by boiling at 100°C for 5‒10 min. Proteins were separated on a 4%–12% SDS-PAGE gel (ACE Biotechnology, Changzhou, China) and then transferred to a nitrocellulose PVDF membrane (Millipore, Shanghai, China). After transfer, membranes were blocked with Rapid Protein-Free Blocking Solution (ACE Biotechnology) at room temperature for 30 min. Membranes were incubated overnight at 4°C with specific primary antibodies, followed by incubation with horseradish peroxidase-conjugated secondary antibodies (Proteintech, Wuhan, China) for 1 h at room temperature. Protein bands were visualized using an ultra-sensitive ECL chemiluminescent reagent (P10100, NCM Biotech, Suzhou, China) and analyzed with ImageJ. Each band intensity was normalized to glyceraldehyde-3-phosphate dehydrogenase (GAPDH). The antibodies used in this study included anti-rabbit HA tag (51064-2-AP, Proteintech), anti-rabbit LC3B (14600-1-AP, Proteintech), anti-rabbit StAR (A1035, ABclonal, Wuhan, China), anti-rabbit GAPDH (AC027, ABclonal), anti-rabbit p62 (T55546S, Abmart, Shanghai, China), and anti-rabbit FSHR (22665-1-AP, Proteintech). All antibodies have been validated for specificity to chicken proteins, including LC3 (Zhu et al., 2024), StAR (Yu et al., 2025), FSHR (Sun et al., 2020), p62 (Tu et al., 2024), and GAPDH (Chen et al., 2025). Protein expression levels were normalized to GAPDH and are presented as fold changes relative to the control group.

### Real-time quantitative PCR

Total RNA was isolated from GCs using a total RNA isolation kit (RC112, Vazyme, Nanjing, China) following the manufacturer's instructions. RNA concentration and purity were measured using a NanoDrop spectrophotometer (Thermo Fisher Scientific, MA, USA). Only RNA samples with an A260/A280 ratio between 1.8 and 2.1 were used for subsequent analyses. RNA integrity was assessed by agarose gel electrophoresis. For cDNA synthesis, a total of 1 μg RNA was reverse transcribed using the Evo M-MLV RT Mix Kit (AG11728, Accurate Biology, Changsha, China) following the manufacturer's protocol. The resulting cDNA was diluted and used as the template for quantitative real-time PCR (qRT-PCR). qRT-PCR was performed on a Bio-Rad CFX96 Real-time Detection System (Bio-Rad) using the SYBR Green Pro Taq HS system (AG11701, Accurate Biology). The amplification program was as follows: initial denaturation at 95°C for 30 s, followed by 40 cycles of denaturation at 95°C for 5 s and annealing at 60°C for 30 s. A melting curve analysis (65–95°C) was conducted at the end of each run to verify amplification specificity. All exon-spanning primers were designed using Primer-BLAST based on chicken gene sequences available in the NCBI database and synthesized by Sangon Biotech (Shanghai, China). Primer sequences are provided in Table 2. *β-actin* was used as the internal reference gene. Relative gene expression levels were calculated using the 2^−ΔΔCt^ method (Livaka and Schmittgenb, 2001). Each sample was analyzed in triplicate technical replicates, and three independent biological replicates were included for each experimental group.

**Table 2:** qRT-PCR primers sequence of related genes.

Gene	Primer Sequence (5’-3’)	Product Length (bp)	Accession No.
*β-actin*	F: CAGCCATCTTTCTTGGGTAT R: CTGTGATCTCCTTCTGCATCC	169	NM_205518.1
*GSK3β*	F:GGAGCCACCGATTATACCTCC R: TGTAGGCGTTCCCAGAACCTTG	144	XM_052666199.1
*FSHR*	F:GAAAGTCTTCCAAGGAGCCA R:TCCGATTTGTAAATGCACAGC	212	NM_205079.2
*AMH*	F:CCCCTCTGTCCCTCATGGA R:CGTCATCCTGGTGAAACACTTC	71	XM_046933682.1
*LHCGR*	F:GAGCTGTGTGACAACTTGCG R:TTGAAGGCATGGCTGTGGAT	125	NM_204936.2
*PCNA*	F:GATGTTCCTCTCGTTGTGGAG R:TCCCAGTGCAGTTAAGAGCC	107	NM_204170.3
*CDK2*	F:ACGTGATCCACACGGAGAAC R:GCAGCTGGAACAGGTAGCTC	132	NM_001199857.2
*CYP11A1*	F: TCCGCTTTGCCTTGGAGTCTGTG R: ATGAGGGTGACGGCGTCGATGAA	112	NM_001001756.2
*StAR*	F: GGTGGACAACATGGAGCAGA R: GAGCACCGAACACTCACAAA	159	NM_204686.3
*ESR1*	F:ACCAACCTTGCAGACAGAGA R:CTAACCAGGCACATTCCAGC	115	NM_205183.2
*PGR*	F:AGCTTTGAATCGCTACCCCA R:TGCCCTTCCATTGCCCTTTT	122	NM_205262.2
*LC3/MAP1LC3B*	F: CAAGAGTCTGGCCACCCG R:CTTGTGTAGCAGTCAGAGCCA	203	XM_419549.8
*P62/SQSTM1*	F:TGCACCCCAACGTCATCTG R:TGTGGATGCCTTTACCCTCG	117	XM_004944954.5
*ATG3*	F:CACTCCCGTCCTCAAGGAGT R:ATCCCCCATCACCATCGTCT	263	NM_001278070.2
*ATG5*	F:GAGGGGTGCTTTCAGTTCCA R:TGAAGCAGGTTGGTATGCGT	129	NM_001006409.2
*ATG10*	F: AGAGGAGGCTCAGATGTCCTG R: CACTCCCAGCCATCCCCTAT	126	XM_046905905.1
*ULK1*	F: AAGCTGGTAGCAATGACAGCC R:CTGGAAGACCCCACTGAAAAC	208	XM_415091.8
*BECN1*	F:GAGCAGGAAGAAGCTCAGTATCA R:AACGCATCTGGTTCTCCACA	100	NM_001006332.1
*NPC1*	F: TGGCGTGGGAAAAGGAGTTT R: CTCAGGACGATCAGGATGCC	256	XM_419162.8
*NPC2*	F:CTGCGGTTCCAAAGACGGA R:CTTGGCTCTCGATCTTGCTGG	128	NM_001031203.2
*POR*	F: ATGAAGAAGACGGGCCGAAA R: TCTTTGGAGAGGCGATTGGC	86	NM_001195796.2
*MSMO1*	F: AGTGGGAAGAGATGCCTAGGT R: AGTGCCAGGCATCCTCAATC	78	XM_046940055.1
*SREBF1*	F:CGCTACCGCTCATCCATCAA R:AGGATCGCCGACTTGTTGAG	89	NM_204126.3
*SREBF2*	F:GGACTTCCCGGACCTGTTTT R:GCTGGATGATCCTGGTCTGG	305	XM_015289037.4
*NR0B1*	F: GCTCTTCAACCCGGATCTACC R: AGAGTTCCTTTGCATTGGATTTATT	303	NM_204593.2
*caspase 3*	F: GCTCTGGTCCTCAGAAAGGG R: GCAGGTAAGTTCATTCCTTTGGA	269	XM_046915475.1
*caspase 6*	F: AAGGCTGCCAGATAGACGTG R: TAGTCATCCCGAGAGGCTTCA	147	NM_001396146.1
*caspase 7*	F: TCATAGGCAAACCCAAGCTGT R: TCCAGGAATAATAACCTGGCACT	186	XM_025151842.3
*LDLR*	F: GCTGGCGGTGCTGCTGCCTTTA R: TGGTGCCCTCCGTGCTCCTT	138	NM_204452.1
*HMGCR*	F: TCAGGAGCGAGGAGTGTC R: TTTGTTATCCAGGTATAGTGGT	187	NM_204485.3
*SCARB1*	F: GTGAACTACTGGCGGACCAA R: AGTGTCATGGATCTGCAGGC	125	XM_015275627.4

### RNA-seq library preparation and transcriptome analysis

Total RNA was extracted from cultured GCs treated with OE-GSK3β or an empty vector for 48 h. RNA concentration and purity were measured using a NanoDrop spectrophotometer, and RNA integrity was evaluated with an Agilent 2100 Bioanalyzer. mRNA was enriched from total RNA with mRNA Capture Beads and fragmented by high-temperature treatment. The fragmented mRNA was then used to construct cDNA libraries for sequencing on the Illumina NovaSeq X Plus platform. Raw reads underwent quality control with fastq (Chen et al., 2018) to obtain clean data. Clean reads were aligned to the reference genome using HISAT2 (Kim et al., 2015), and gene expression levels were quantified with FPKM. Differentially expressed genes (DEGs) were identified by edgeR (Robinson et al., 2010) with thresholds of *P*-Value < 0.05 and |log_2_ (Fold Change)| > 1. Finally, KEGG and Reactome pathway enrichment analyses were performed on the DEGs. DEGs involved in the autophagy and lipid metabolism pathways were validated using qRT-PCR. Primer sequences for these DEGs are listed in Table 2.

### GFP-LC3 assay

GCs were transfected with an overexpression vector or siRNAs at the time of transfection with the GFP-LC3 plasmid, which is maintained in our laboratory. Briefly, after treatment, cells were fixed with 4% paraformaldehyde for 30 min. Subsequently, random images were captured using an IX73P1F microscope, which was used to take pictures randomly following the procedures described previously (Han et al., 2023).

### Total cholesterol and malondialdehyde assay

After treatment, cells and supplements were collected for total cholesterol (T-CHO) quantification using a T-CHO assay kit (A111-1-1, Jiancheng), following the manufacturer's instructions. Briefly, the culture supernatants were collected post-treatment and used directly for T-CHO determination. For intracellular T-CHO measurement, GCs were washed with PBS and lysed using cell lysis buffer. The lysates were then collected and centrifuged, and the resulting supernatants were used for T-CHO analysis. Protein concentration of each sample was determined using a BCA protein assay kit for normalization.

The levels of malondialdehyde (MDA) in both the supernatants and cells were measured in the combination treatment of OE-GSK3β and Rapa, following the manufacturer's protocol (A003-1-2, A003-4-1, Jiancheng).

### Lipid droplet analysis by Oil Red O staining and BODIPY

Lipid droplets in GCs were stained with Oil Red O. After treatment, the cells were washed with PBS and fixed with 4% paraformaldehyde for 10 min. Subsequently, the cells were stained with Oil Red O Stain Kit (G1262, Solarbio, Beijing, China) according to the manufacturer's instructions and washed with PBS twice. Finally, images were captured using an IX73P1F microscope. The percentage of Oil Red O-positive area was quantified using ImageJ.

Cells were also stained with BODIPY 493/503 (C2053S, Beyotime) to visualize intracellular lipid droplets. Briefly, cultured cells were washed with PBS and fixed with 4% paraformaldehyde for 15 min at room temperature, followed by three washes with PBS. After fixation, cells were incubated with BODIPY solution according to the manufacturer’s instructions for 15 min at room temperature in the dark. Cell nuclei were counterstained with DAPI. After final washes, cells were mounted and imaged using an IX73P1F microscope. Quantification of fluorescence intensity was performed using ImageJ.

### Detection of ATP levels

Intracellular ATP concentration was measured using a commercial ATP assay kit (S0026, Beyotime). Briefly, after removing the medium and washing the cells with PBS, 100 uL of ATP lysis solution was applied to the cells for 15 min with agitation. The lysates were collected and centrifuged at 12,000 × g for 5 min at 4°C. The harvested supernatants were then used for the subsequent ATP quantification assays. ATP levels were determined by a Microplate Reader (Epoch 2). Data were normalized to the control group and are expressed as fold change relative to the control.

### Determination of mitochondrial membrane potential

To investigate whether mitochondrial membrane potential (MMP) is affected by lipid accumulation upon GSK3β or Rapa treatment, MMP was determined using the Mito-Tracker Red CMXRos kit (C1049, Beyotime). Briefly, after treatment, Mito-Tracker Red CMXRos was added to the medium at a concentration of 100 nM and incubated for 30 min at 37°C. Cells were then washed and stained with DAPI. Fluorescence images were captured using an IX73P1F microscope. Fluorescence intensity was quantified using ImageJ.

### Statistical analysis

All experiments in this study were conducted with at least three independent biological replicates, each containing at least three technical replicates. Statistical analyses were performed using SPSS 20.0 (IBM Corp., Armonk, NY, USA). Tests for homogeneity of variance were conducted prior to analysis of variance. Differences between the two groups were analyzed using Student’s t-test, while multiple comparisons were performed using one-way analysis of variance (ANOVA) followed by Tukey's post hoc test. Data are presented as means ± SEM and visualized using GraphPad Prism 9.0 (San Diego, CA, USA). For all comparisons, ns indicates no significance; * *P* < 0.05 between groups. Different letters denote significant differences among groups.

## Results

### GSK3β inhibits granulosa cell differentiation and promotes steroidogenesis

**Fig. 1:**
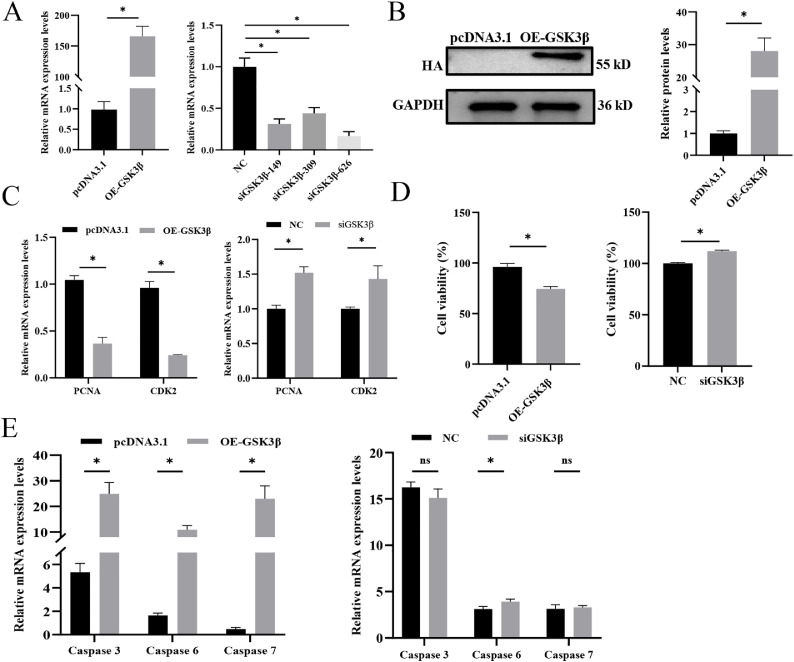
GSK3β inhibits the proliferation of granulosa cells from prehierarchical follicles in chickens. (A) and (B). Overexpression and knockdown efficiency were confirmed after transfection with OE-GSK3β (pcDNA3.1-HA-GSK3β) and siRNA using qRT-PCR and Western blot. (C). Relative mRNA expression levels of proliferation marker genes, (D) cell viability, and (E) Relative mRNA expression levels of apoptosis marker genes following overexpression or knockdown of GSK3β. *P < 0.05, vs Empty vector or Negative control. PCNA: Proliferating Cell Nuclear Antigen; CDK2: Cyclin Dependent Kinase 2; GAPDH: Glyceraldehyde-3-Phosphate Dehydrogenase; Caspase: Cysteine-dependent aspartate-specific protease.

To examine the impact of GSK3β on GC function, we conducted transfections using OE-GSK3β and specific siRNAs. As illustrated in Fig. 1A, B, the cells transfected with OE-GSK3β exhibited a significant increased GSK3β mRNA and protein levels compared to the control group (*P* < 0.01). In contrast, transfection with siRNAs led to a marked reduction in GSK3β mRNA expression (*P* < 0.05), with siGSK3β-626 producing the most substantial effect (*P* < 0.05). Therefore, siGSK3β-626 was employed in all subsequent experiments. Further investigation into cell proliferation revealed that GSK3β overexpression significantly decreased mRNA expression of proliferation marker genes (*PCNA* and *CDK2*) (*P* < 0.05), whereas GSK3β knockdown significantly increased their expression levels (*P* < 0.05) (Fig. 1C). Similarly, cell viability was significantly reduced in cells overexpressing GSK3β (*P* < 0.01) but significantly enhanced following GSK3β knockdown (*P* < 0.05) (Fig. 1D). Apoptosis of GCs was also assessed following GSK3β overexpression or knockdown. As shown in Fig. 1E, the mRNA expression levels of apoptosis markers (*Caspase3, Caspase6, Caspase7*) were significantly elevated by GSK3β overexpression (*P* < 0.05); in contrast, their expression remained largely unaltered upon GSK3β knockdown, except for *Caspase6*.

**Fig. 2:**
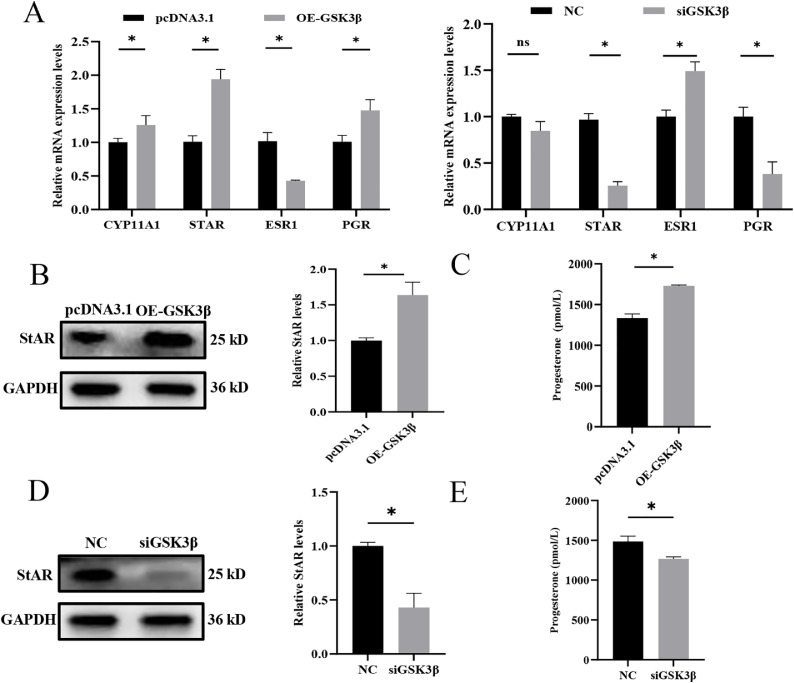
GSK3β promotes progesterone synthesis of granulosa cells from prehierarchical follicles in chickens. (A). Relative mRNA expression levels of steroid hormone synthesis following GSK3β overexpression and knockdown using qRT-PCR. (B). Levels of steroid hormone synthesis enzyme of StAR follwing GSK3β overexpression. (C). Contents of progesterone were measured following GSK3β Overexpression. ns = no significant; (D) StAR protein levels, and (E) progesterone levels folowing knockdown GSK3β. *P < 0.05. CYP11A1: Cytochrome P450 Family 11 Subfamily A Member 1; StAR: Steroidogenic Acute Regulatory Protein; ESR1: Estrogen Receptor 1; PGR: Progesterone Receptor; GAPDH: Glyceraldehyde-3-Phosphate Dehydrogenase.

Upon GSK3β overexpression, the mRNA expression levels of steroid hormone synthesis marker genes (*CYP11A1, StAR, ESR1*, and *PGR*) in GCs were significantly elevated (*P* < 0.05) (Fig. 2A). This finding was corroborated by Western blot analysis, which showed a substantial significant increase in StAR protein levels (*P* < 0.05) (Fig. 2B). Additionally, the concentrations of progesterone in the culture medium were significantly higher following GSK3β overexpression (*P* < 0.05) (Fig. 2C). Conversely, these indicators exhibited opposite changes following GSK3β knockdown (Fig. 2).

**Fig. 3:**
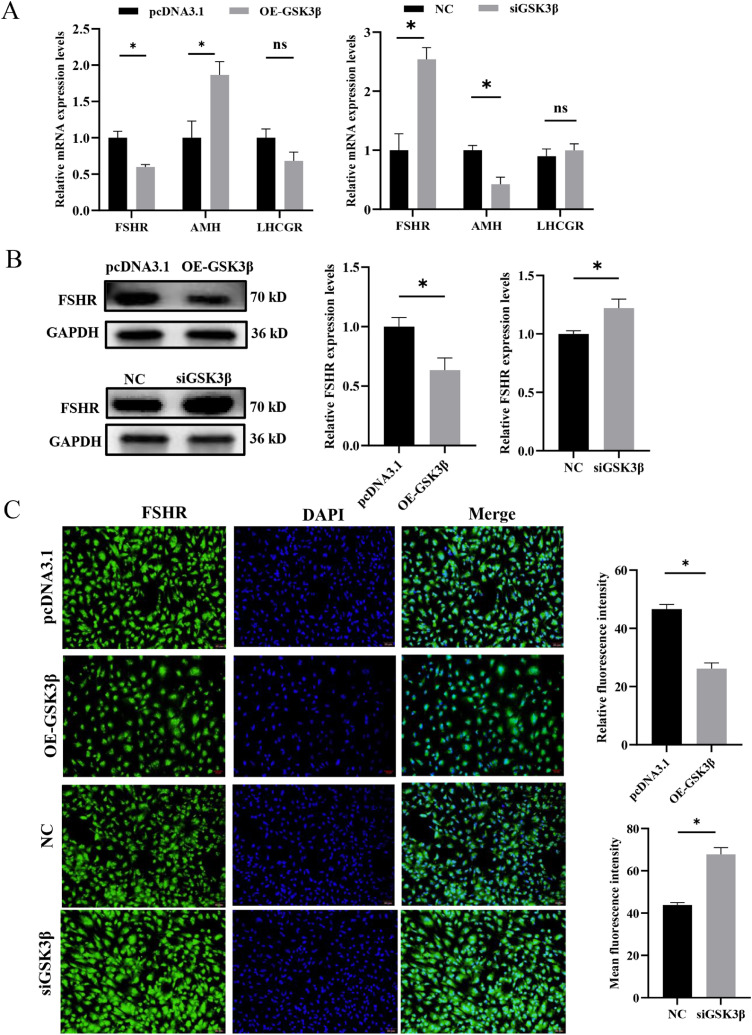
GSK3β inhibits differentiation of granulosa cells from pre-hierarchical follicles in chickens. (A). Relative mRNA expression levels of differentiation marker genes; (B). Western blot and (C). immunofluorescence analysis of FSHR protein levels following treatment with GSK3β overexpression and knockdown. Scale bars represent 50 μm. ns = no significant; *P < 0.05. FSHR: Follicle Stimulating Hormone Receptor; AMH: Anti-Mullerian Hormone; LHCGR: Luteinizing Hormone/Choriogonadotropin Receptor; GAPDH: Glyceraldehyde-3-Phosphate Dehydrogenase.

In the context of differentiation, quantitative analysis demonstrated a significant reduction in *FSHR* mRNA expression (*P* < 0.05) and a significant increase in *AMH* mRNA following the overexpression of GSK3β (*P* < 0.05), while a slight decrease in *LHCGR* but no significant difference (Fig. 3A). These results were corroborated by both western blot and immunofluorescence analyses, which confirmed a substantial decrease in FSHR protein levels upon GSK3β overexpression (*P* < 0.05) (Fig. 3B and C). Conversely, the knockdown of GSK3β elicited opposite effects across all observed genes and proteins.

Collectively, these findings demonstrate that GSK3β suppresses proliferation and differentiation in GCs, while simultaneously promoting steroid hormone production and apoptosis. Therefore, the molecular mechanisms underlying this phenomenon warrant further investigation.

### Transcriptomic analysis reveals the regulatory role of GSK3β in granulosa cell autophagy and lipid metabolism

**Fig. 4:**
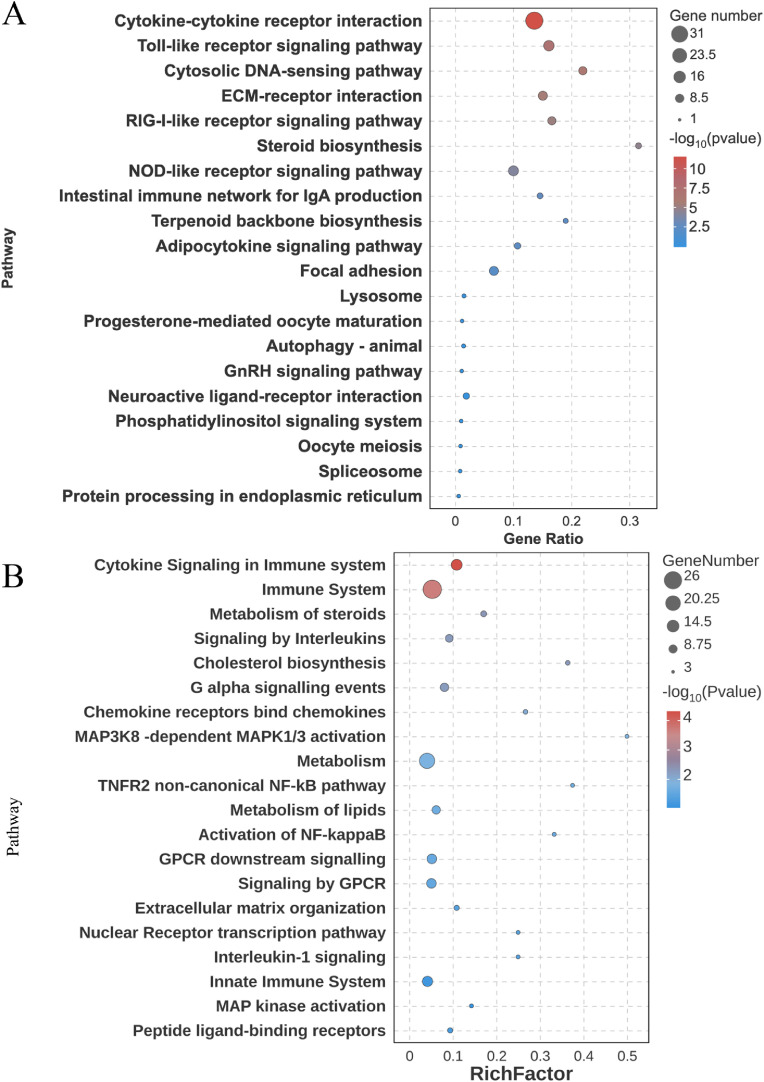
RNA-Seq Analysis of the pathways enrichment involved in overexpression GSK3β in granulosa cells. (A). KEGG pathways enriched with differentially expressed genes upon overexpression GSK3β. (B). Reactome analysis of enriched pathways for differentially expressed genes the following GSK3β expression.

To elucidate the molecular mechanisms through which GSK3β modulates GC function, we conducted RNA-seq analysis on GCs overexpressing GSK3β. All raw sequencing data have been deposited in the GSA database (Accession number: CRA030526). Differential expression analysis identified 860 differentially expressed genes (DEGs), comprising 708 upregulated and 152 downregulated DEGs (Fig. S1). KEGG pathway analysis indicated that these DEGs are involved in various signaling pathways, including Cytokine-cytokine receptor interaction, ECM-receptor interaction, steroid biosynthesis, lysosome, and autophagy signaling pathways (Fig. 4A). Furthermore, Reactome pathway enrichment analysis revealed significant enrichment of DEGs in multiple pathways related to metabolism of steroids, metabolism, and metabolism of lipids (Fig. 4B).

**Fig. 5:**
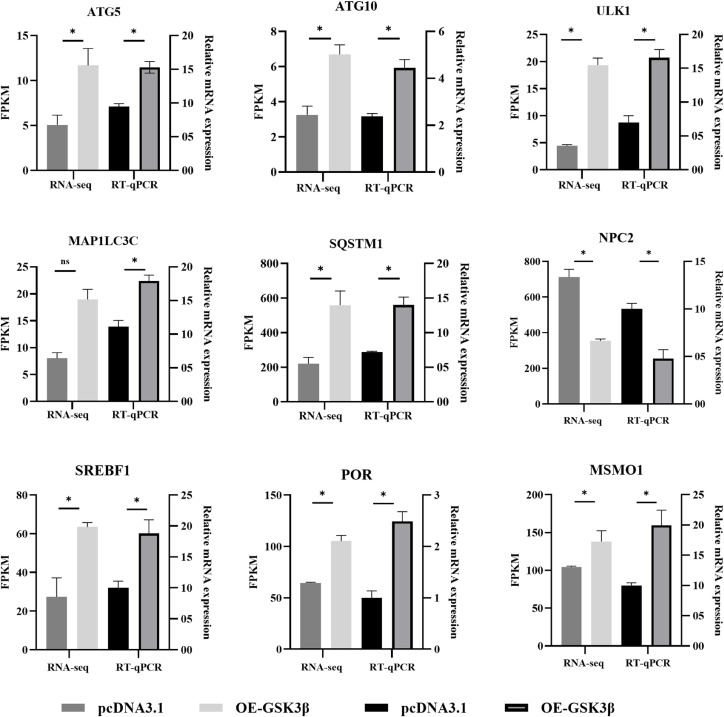
qRT-PCR validation of differentially expressed genes associated with autophagy and lipid metabolism pathways. ns = no significant; *P < 0.05. ATG5: Autophagy Related 5; ATG10: Autophagy Related 10; ULK1: Unc-51 Like Autophagy Activating Kinase 1; MAP1LC3B: Microtubule Associated Protein 1 Light Chain 3 Beta; SQSTM1: Sequestosome 1; NPC2: NPC Intracellular Cholesterol Transporter 2; SREBF1: Sterol Regulatory Element Binding Transcription Factor 1; POR: Cytochrome P450 Oxidoreductase; MSMO1: Methylsterol Monooxygenase 1.

The autophagy signaling pathway has emerged as a pivotal regulator of GC function. Given that GSK3β is known to induce autophagy (Ryu et al., 2021) and serves as a central hub integrating energy metabolism and the Wnt signaling pathway (Beurel et al., 2015), we focused on this pathway to explore whether GSK3β affects GC function through an autophagic mechanism. To validate the accuracy of the sequencing results, nine DEGs associated with autophagy and lipid metabolism were randomly selected for qRT-PCR analysis. The expression trends observed in qRT-PCR were highly consistent with the RNA-seq data (Fig. 5), confirming the reliability of the transcriptomic sequencing results.

**Fig. 6:**
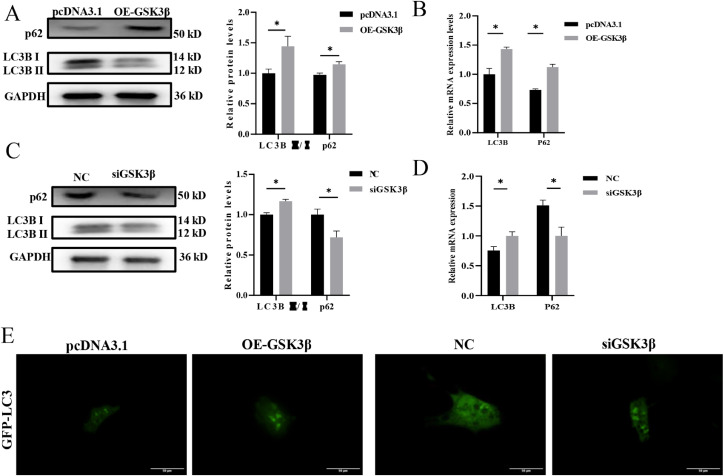
GSK3β induces autophagosome accumulation in granulosa cells. The protein (A, C), and mRNA (B, D) expression levels of LC3 and p62 in granulosa cells after transfection with OE-GSK3β and siRNA. (C). Representative images of GFP-LC3 in the empty vector group, the GSK3β overexpression group, the negative control group, and the GSK3β knockdown group. GFP-LC3 (green) spots are autophagosomes (scale bars represent 50 μm). ns = no significant; *P < 0.05. LC3B: Microtubule-associated protein 1 light chain 3B; p62: Sequestosome-1; GAPDH: Glyceraldehyde-3-Phosphate Dehydrogenase.

To determine whether autophagy mediates the effects of GSK3β on GCs, we examined alterations in the autophagy marker proteins LC3 and p62, as well as the number of autophagosomes. As shown in Fig. 6A–D, the findings indicated a significant increase in LC3 conversion and p62 expression following GSK3β overexpression (*P* < 0.05), whereas p62 expression significantly decreased after GSK3β knockdown (*P* < 0.05). GFP-LC3 analysis demonstrated an increase in the number of autophagosomes in GCs overexpressing GSK3β compared to control groups (Fig. 6E). These observations suggest that both gain- and loss-of-function of GSK3β modulate autophagosome formation in GCs. However, GSK3β overexpression appears to inhibit autophagic flux, leading to the accumulation of autophagic substrates and thereby impeding autophagy.

### GSK3β inhibits granulosa cell differentiation while promoting steroidogenesis, accompanied by a cholesterol metabolism alteration

Overexpression of GSK3β result in decreased FSHR expression and increased StAR expression in GCs, which is notably inconsistent with the well-established knowledge that FSHR and StAR in prehierarchical GCs typically exhibit a synergistic relationship during follicle selection, where elevated FSHR levels promote StAR expression, thereby enhancing steroid hormone synthesis (Francoeur et al., 2023; Zhang et al., 2024a). To investigate the dysregulation between differentiation and steroidogenesis, and based on prior research indicating that abnormal or premature luteinization of follicular GCs is a key factor contributing to follicular atresia, which itself is a significant indicator of reduced FSHR activity (Fan et al., 2010; Owens et al., 2019), as well as the lipid metabolism in Reactome pathways we obtained, we further examined cholesterol metabolism (Schade et al., 2020).

**Fig. 7:**
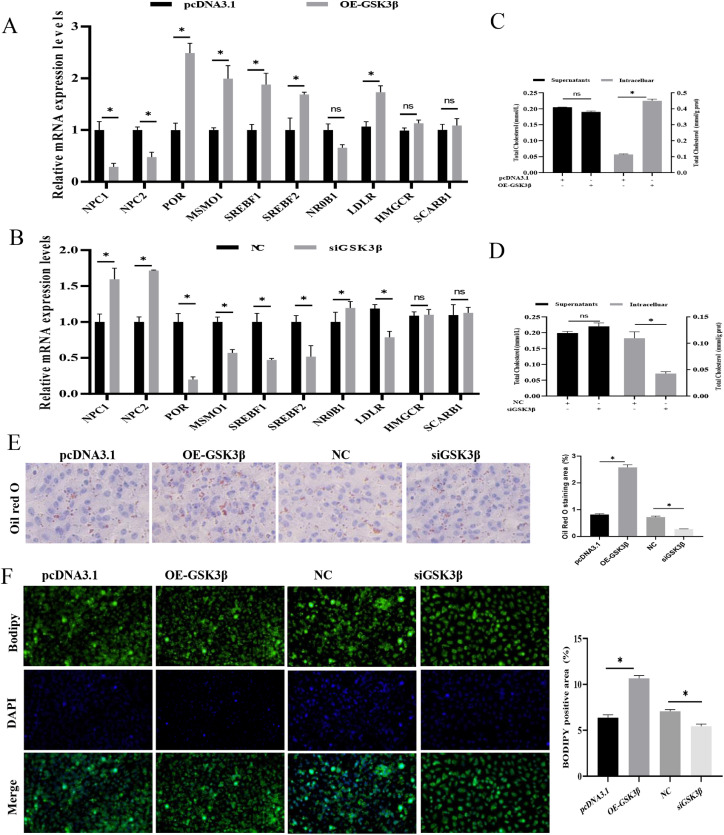
GSK3β promotes lipid accumulation in granulosa cells. (A, B). qRT-PCR showed the transport and synthesis of cholesterol related to genes of granulosa cells following overexpression or knockdown of GSK3β. (C, D). Total cholesterol contents in the supernatants and intracellular of granulosa cells following overexpression or knockdown of GSK3β. (E). Oil red O staining, and (F) BODIPY stainging of each group (scale bars represent 50 μm). NPC1: NPC Intracellular Cholesterol Transporter 1; NPC2: NPC Intracellular Cholesterol Transporter 2; POR: Cytochrome P450 Oxidoreductase; MSMO1: Methylsterol Monooxygenase 1; SREBF1: Sterol Regulatory Element Binding Transcription Factor 1; SREBF2: Sterol Regulatory Element Binding Transcription Factor 2; NR0B1: Nuclear Receptor Subfamily 0 Group B Member 1; LDLR: Low-Density Lipoprotein Receptor; HMGCR: 3-Hydroxy-3-methylglutaryl-CoA reductase; SCARB1: Scavenger Receptor Class B, Member 1; Rapa: Rapamycin.*P < 0.05. Different letters indicate significant difference, while similar letters indicate no difference.

The results revealed that GSK3β overexpression significantly downregulated the expression of cholesterol transport-related genes (*NPC1* and *NPC2*) (*P* < 0.05), while significantly upregulating the expression of cholesterol synthesis-related genes (*MSMO1, SREBF1, SREBF2*) and *POR* (*P* < 0.05), and the lipid uptake related genes (*LDLR*) (Fig. 7A). Knockdown of GSK3β reduced the expression trends of cholesterol transport and synthesis genes (Fig. 7B). The total cholesterol levels in supernatants increased slightly but no significantly, while the intracellular cholesterol content in GCs markedly increased (*P* < 0.05, Fig. 7C, D). Oil O red staining and BODIPY staining both showed a significant rise in lipid droplets (*P* < 0.05, Fig. 7E, F). These findings indicate that GSK3β promotes lipid droplet accumulation.

### Rapamycin counteracts the inhibition of differentiation and promotes progesterone synthesis caused by GSK3β

**Fig. 8:**
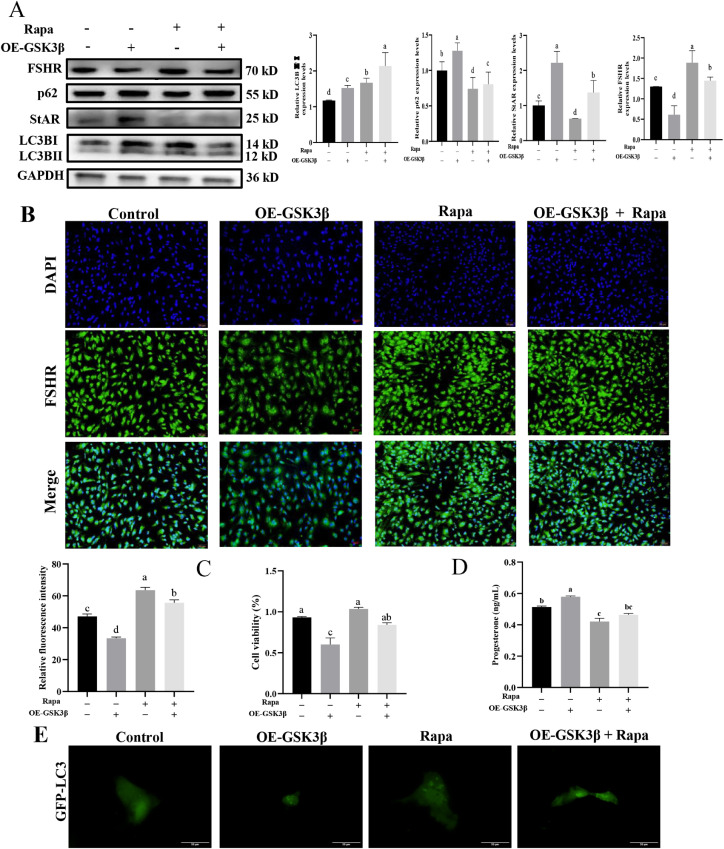
GSK3β inhibits differentiation of granulosa cells through autophagy. (A). Western blot showed the expression levels of LC3B, StAR, p62, and FSHR in the control group, and the treatment groups with the overexpressed GSK3β, 1μM Rapa, and a combination of GSK3β overexpression and Rapa. (B). FSHR expression as detected by immunofluorescence in the control group, GSK3β overexpression group, Rapa group, and combination group (scale bars represent 50 μm). (C). Cell viability was detected in the control, overexpression, Rapa, and combination of overexpression and Rapa groups. (D). Contents of progesterone in granulosa cells of the groups of control, GSK3β overexpression, Rapa, and the combination. (E). Representative images of GFP-LC3 in the control group, OE group, Rapa group, and OE+Rapa group. GFP-LC3 (green) dots are autophagosomes (scale bars represent 50 um). FSHR: Follicle Stimulating Hormone Receptor; StAR: Steroidogenic Acute Regulatory Protein; LC3B: Microtubule-associated protein 1 light chain 3B‌; p62: Sequestosome-1‌; GAPDH: Glyceraldehyde-3-Phosphate Dehydrogenase; Rapa: Rapamycin. Different letters indicate significant difference, while similar letters indicate no difference.

Rapamycin has been shown to be an effective autophagy activator in a previous study (Shao et al., 2022). Therefore, cells were treated with Rapa under conditions of OE-GSK3β. Combined treatment with Rapa and GSK3β restored the decreased FSHR expression levels caused by treatment GSK3β alone, as evidenced by Western blot and immunofluorescence results (*P* < 0.05, Fig. 8A, B), as well as cell viability (Fig. 8C). Additionally, the decreased StAR expression levels and steroid hormone synthesis observed with treatment GSK3β alone were partially restored upon combined treatment (Fig. 8A and D). Notably, GSK3β-induced autophagic flux arrest was rescued by combined Rapa treatment, which increased LC3 conversion while partially eliminating p62 accumulation (Fig. 8A). Furthermore, the accumulation of autophagosomes induced by GSK3β was reduced, as shown by the GFP-LC3 assay (Fig. 8E). These results demonstrate that Rapa rescues GSK3β-induced differentiation inhibition, cell viability decline, and autophagic flux arrest, indicating that GSK3β influences GC function through autophagy.

### GSK3β promotes granulosa cell steroidogenesis by promoting lipid droplet accumulation

**Fig. 9:**
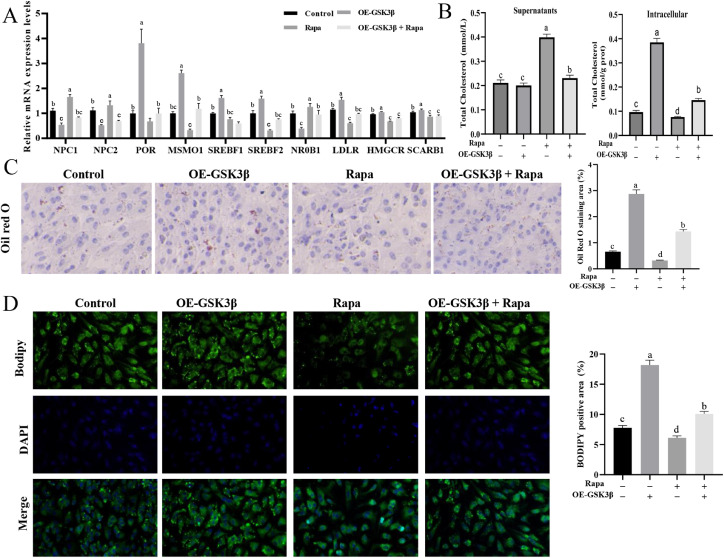
GSK3β promotes steroidogenesis of granulosa cells via compensatory enhancing cholesterol metabolism. (A). qRT-PCR showed the mRNA expression levels of genes related to the synthesis and transport of cholesterol in granulosa cells in the control group, OE group, Rapa group, and OE-GSK3β+Rapa group. (B). Total cholesterol contents in the supernatants and intracellular following treatment with Rapa, GSK3β overexpression, and the combination treatment. (C). Oil red O staining, and (D) BODIPY staining of each group (scale bars represent 50 μm). NPC1: NPC Intracellular Cholesterol Transporter 1; NPC2: NPC Intracellular Cholesterol Transporter 2; POR: Cytochrome P450 Oxidoreductase; MSMO1: Methylsterol Monooxygenase 1; SREBF1: Sterol Regulatory Element Binding Transcription Factor 1; SREBF2: Sterol Regulatory Element Binding Transcription Factor 2; NR0B1: Nuclear Receptor Subfamily 0 Group B Member 1; LDLR: Low-Density Lipoprotein Receptor; HMGCR: 3-Hydroxy-3-methylglutaryl-CoA reductase; SCARB1: Scavenger Receptor Class B, Member 1. Rapa: Rapamycin. *P < 0.05. Different letters indicate significant difference, while similar letters indicate no difference.

The steroid hormone synthesis and cholesterol metabolism were examined upon combination treatment. Results showed that treatment Rapa alone significantly influenced the expression levels of lipid synthesis and transport related genes (Fig. 9), while combined treatment with Rapa and GSK3β, which counteracted the effects of OE-GSK3β, leading to the alterations in the expression of lipid synthesis and transport-related genes (Fig. 9A). Total cholesterol levels in the supernatants increased significantly upon Rapa treatment but decreased with the combination treatment, while intracellular total cholesterol levels decreased significantly compared to treatment OE-GSK3β alone (*P* < 0.05, Fig. 9B). Oil Red O staining further demonstrated that GSK3β overexpression led to a significant increase in intracellular lipid droplets, which was significantly decreased after combined Rapa treatment (*P* < 0.05, Fig. 9C). BODIPY staining further confirmed the effects of OE-GSK3β on lipid accumulaiton (Fig. 9D). These findings indicate that GSK3β promotes lipid accumulation by inducing autophagy arrest, leading to a compensatory enhancement of cholesterol synthesis and lipid uptake in GCs and subsequent excessive steroid hormone production.

### GSK3β inducess granulosa cell lipid accumulation accompanied by mitochondrial function damage

**Fig. 10:**
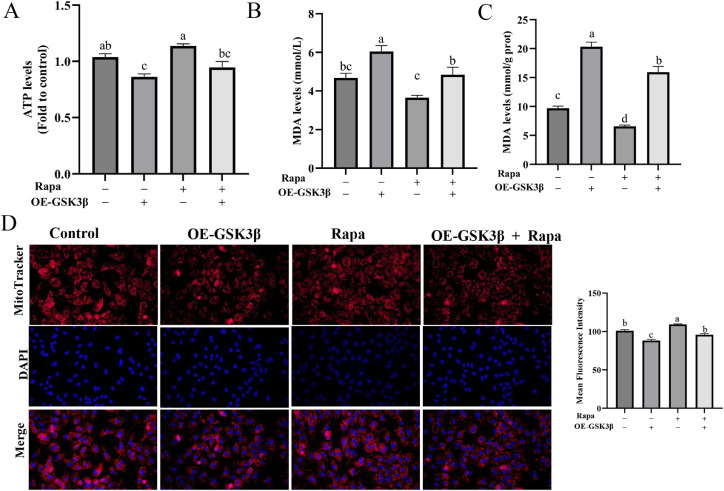
GSK3β induced granulosa cells lipid accumulation accompanied by mitochondrial function damage. (A) Intracellular ATP levels relative to the control group. (B, C) MDA levels measured in supernatants (B) and intracellular (C). (D) Representative immunofluorescence images of mitochondrial membrane potential using MitoTracker Red. Scale bar = 50 µm. Quantitative analysis of the mean fluorescence intensity is shown on the right. Different letters indicate statistically significant differences (*P* < 0.05). OE-GSK3β, GSK3β overexpression; Rapa, rapamycin.

Based on the above findings, we hypothesized that excessive lipid accumulation may impair mitochondrial function. To test this hypothesis, ATP levels, the lipid peroxidation marker MDA, and MMP were assessed. The results demonstrated that GSK3β overexpression decreased ATP levels, whereas Rapa treatment increased ATP levels. Co-treatment with Rapa and OE-GSK3β partially rescued the ATP reduction induced by OE-GSK3β, although the difference was not statistically significant (Fig. 10 A). MDA assays showed that OE-GSK3β significantly increased both intracellular and supernatant MDA levels (*P* < 0.05), with a more pronounced elevation observed intracellularly. Co-treatment with Rapa reduced MDA levels (Fig. 10 B, C). MMP analysis revealed that OE-GSK3β significantly decreased MMP, while co-treatment with Rapa markedly restored MMP levels (*P* < 0.05, Fig. 10D). These results suggest that excessive lipid accumulation induced by OE-GSK3β leads to mitochondrial damage to a certain extent, which may be an important mechanism underlying the inhibition of GC differentiation.

## Discussion

Improving poultry egg production depends on the orderly progression of follicle selection, in which GCs play a crucial role in determining follicle fate. The expression levels of FSHR in GCs determine their ability to effectively respond to FSH, thereby enabling their selection for progression to the hierarchical stage (Johnson, 2015; Zhou et al., 2013). Following the specific binding of FSH to FSHR, activation of the cAMP/PKA signaling pathway triggers a cascade that promotes rapid differentiation and proliferation of GCs. This transformation drives a shift toward hormone-synthesizing dominant cells (Johnson, 2015; Zhang et al., 2024a). Therefore, investigating the changes that influence the coordination of GC differentiation, proliferation, and hormone synthesis is of great significance for research on follicle selection. Nonetheless, the precise mechanisms regulating differentiation remain poorly understood. Previous research has suggested that GSK3β, which is responsive to FSH, may affect follicular development (Dong et al., 2022a). Our findings reveal that GSK3β inhibits the differentiation and proliferation of GCs while simultaneously promoting steroidogenesis by enhancing lipid accumulation via the autophagy pathway. This discovery indicates that GSK3β acts as a potential suppressor of GC differentiation. Overall, the regulatory role of GSK3β in GCs involves alterations in multiple cellular functions, reflecting a complex interplay between the control of cellular differentiation and hormone synthesis.

As a central mediator of cellular crosstalk, GSK3β is involved in multiple signaling pathways, including Wnt, AMPK, and mTOR, rendering it one of the most versatile kinases (Beurel et al., 2015; Hoffmeister et al., 2020). Concurrently, these pathways regulate changes in ovarian function by influencing GSK3β. For example, GSK3β has been shown to influence steroid hormone production in theca cells during human premature ovarian failure by regulating mitochondrial dynamics (Xiong et al., 2024), and it suppresses the cellular responsiveness of murine GC to FSH through autophagy (Bérubé et al., 2024). In chickens, FSH delays GC senescence by inhibiting GSK3β (Dong et al., 2022a), whereas phosphorylation of GSK3β at Tyr216 promotes estrogen synthesis in theca cells (Zhang et al., 2024b). Building upon these diverse functional roles, our present study found that GSK3β overexpression suppressed FSHR expression while promoting *AMH* mRNA expression. An expression profile characterized by low FSHR and high AMH typically defines prehierarchical follicles. Conversely, an increase in FSHR coupled with a rapid decline in AMH indicates that a dominant follicle is undergoing selection into the hierarchical stage (Johnson, 2015; Johnson et al., 2008). AMH regulates follicular development in a concentration-dependent manner (Huang et al., 2021), and excessive AMH supplementation in hen diets has been shown to suppress follicle selection (Johnson et al., 2009). Similarly, elevated AMH levels in human ovarian hypoplasia inhibit GC differentiation and induce the premature formation of the follicular corpus luteum complex, leading to ovulatory dysfunction (Man et al., 2022). Therefore, the GSK3β-mediated downregulation of FSHR and upregulation of AMH clearly indicate the suppression of follicle selection. In addition to FSHR and AMH, cell proliferation changes also revealed that GSK3β inhibits cell proliferation and promotes apoptosis. These results further indicate that GC differentiation is inhibited by GSK3β. However, steroid regulation appears inconsistent.

A fundamental event during follicle selection is the acquisition of steroidogenic capacity by GCs (Johnson et al., 2002). Conventionally, enhanced GC differentiation and steroidogenesis are positively correlated (Li et al., 2022a). However, our study reveals a paradoxical scenario induced by GSK3β: suppression of GC differentiation and proliferation occurred concurrently with a significant upregulation of progesterone and its rate-limiting enzyme, StAR. This apparent contradiction—differentiation inhibition alongside elevated StAR and progesterone levels—prompted us to investigate the underlying mechanisms.

Transcriptome analysis following GSK3β overexpression confirmed the involvement of autophagy-related pathways. However, further protein-level analysis revealed that GSK3β primarily inhibits autophagic flux, as evidenced by the accumulated LC3-II /I ratio and p62, indicating blocked autophagic flux (Shao et al., 2022). Previous studies have shown that autophagic flux blockage impairs lysosomal degradation (Johansen and Lamark, 2011; Kumariya et al., 2021) and is associated with suppressed FSHR expression and steroidogenesis (Dong et al., 2022a; Ren et al., 2023; Zhou et al., 2019). Crucially, the impaired autophagic flux and the subsequent reduction in FSHR expression induced by GSK3β were rescued by co-treatment with the autophagy inducer Rapa, confirming that GSK3β disrupts GC differentiation through autophagy blockade. The underlying mechanism warrants further study.

Further Reactome pathway enrichment analysis revealed that GSK3β significantly modulates lipid metabolism. This finding was firmly confirmed by total cholesterol assays, Oil Red O staining, and BODIPY staining, all of which demonstrated an elevated lipid deposition. Autophagy plays a crucial role in cellular lipid homeostasis by delivering intracellular lipid droplets to lysosomes for degradation and turnover (Singh et al., 2009). Inhibition or loss of autophagic flux inevitably results in an elevation of intracellular lipid droplet levels (Ouimet et al., 2011). A pivotal question arising from these findings is how this excessive lipid accumulation leads to cellular dysfunction. We further found that GSK3β overexpression caused a significant rise in MDA levels, a decrease in ATP, and a loss of MMP, all of which were rescued by Rapa. MDA serves as a direct indicator of lipid peroxidation and membrane damage (Hong et al., 2026), and its accumulation is known to impair ATP synthase by disrupting the mitochondrial respiratory chain (Niki, 2009). When autophagic flux is blocked, the massive accumulation of lipid droplets triggers lipotoxicity (Olzmann and Carvalho, 2019), which directly damages mitochondria, disrupting mitochondrial homeostasis. Mitochondria are not only the energetic hubs of the cell but also the structural foundations for GC maturation and steroidogenesis. The differentiation of GCs demands substantial ATP and intact mitochondrial membranes. Therefore, the mitochondrial collapse caused by GSK3β-mediated lipotoxicity deprives the cells of the energy required for differentiation, driving the observed differentiation block. The ability of Rapa to restore mitochondrial parameters confirms that this damage originates from autophagy-dependent lipid overload.

The impact of lipid deposition on progesterone synthesis in GCs varies across species. In bovine corpus luteum GCs, increased lipid droplet levels negatively affect progesterone production (Seekford et al., 2025), whereas in goose GCs, elevated lipid droplet levels enhance progesterone secretion (Hu et al., 2020). In the present study, lipid overload compromises mitochondrial function and thereby inhibits differentiation, while simultaneously creating a condition that favors progesterone overproduction. StAR is the primary rate-limiting enzyme that determines the rate of cholesterol—the precursor for steroid hormone synthesis—from the outer to the inner mitochondrial membrane (Stocco, 2001). Excessive lipid accumulation resulting from blocked lipophagy provides an abundant cholesterol reservoir (Ouimet et al., 2011; Singh et al., 2009). Our data show that GSK3β overexpression leads to dysfunction of genes related to cholesterol transport and synthesis, accompanied by a significant rise in intracellular total cholesterol, which contributes to cellular dysfunction and apoptosis. Upon autophagy activation with Rapa, these effects were reversed, indicating that the cholesterol accumulation depends on autophagic inhibition. Intracellular cholesterol replenishment originates from two primary pathways: extracellular uptake and de novo synthesis within the cell (Jia et al., 2024; Kunkel et al., 2023). In addition to its role as an autophagy agonist, Rapa specifically inhibits the mTOR pathway (Battaglioni et al., 2022), which catalyzes SREBP phosphorylation to promote cholesterol synthesis and uptake (Geng and Guo, 2024; Ma et al., 2013; Panwar et al., 2023; Radulovic et al., 2022). Consequently, the GSK3β-induced autophagic block impairs lipophagy, leading to lipid accumulation. The excess cholesterol substrate—combined with the concurrent rise in StAR, which depends on mitophagy turnover (Koganti et al., 2025)—drives abnormally high progesterone production. Rapa counteracts this effect both by restoring autophagic lipid clearance and by inhibiting mTOR-driven cholesterol accumulation, thereby promoting the aberrant steroidogenic output.

Overall, our results demonstrate that GSK3β-induced blockade of autophagic flux leads to excessive lipid accumulation, which exerts dual consequences. On one hand, lipid overload induces mitochondrial lipotoxicity, depriving GCs of the energy and mitochondrial integrity essential for differentiation. On the other hand, the accumulation of cholesterol and sustained StAR expression drives a paradoxical elevation of progesterone. Therefore, GSK3β-mediated lipid overload is a key mechanism that simultaneously suppresses GC differentiation and enhances progesterone synthesis, resolving the initial paradox observed in this study.

## Conclusion

In conclusion, this study demonstrates that the overexpression of GSK3β inhibits both the differentiation and proliferation of GCs, while simultaneously enhancing cellular progesteone synthesis. In contrast, the knockdown of GSK3β results in the opposite effects. This phenomenon is primarily attributed to the induction of autophagy by GSK3β overexpression, along with a concurrent blockage of autophagic flux. The obstruction of autophagic flux leads to lipid accumulation, which induces mitochondrial damage, thereby disrupting cellular homeostasis. This disruption results in diminished proliferation and aberrant steroidogenesis in GCs, ultimately suppressing differentiation. These findings not only expand our understanding of the regulatory functions of GSK3β in GCs but also elucidate its mechanism of action, providing novel theoretical insights into poultry follicle selection.

## Data and model availability statement

Data was deposited in the GSA database (Accession number: CRA030526).

## Ethics approval

All the procedures involving hens followed the guidelines formulated by the Ministry of Agriculture of the People's Republic of China, with approval from the Animal Care and Use Committee of Jiangsu University of Science and Technology (No. G2025SJ21, Zhenjiang, China).

## Financial support statement

This work was supported by the National Key Research and Development Program of China (2022YFD1300100) and China Agriculture Research Systems (CARS-39-K01).

## Declaration of generative AI and AI-assisted technologies in the writing process

No generative AI or AI-assisted tools were used in any stage of this research or manuscript preparation.

## Author Contributions

**Yuechen Liao** was primarily responsible for the conception and execution of the study, including experimental design, manuscript preparation, data organization, and interpretation of results. **Yuechen Liao** also contributed to the initial drafting and revision of the manuscript. **Ashi Li** contributed to the experimental implementation, data collection, analysis, interpretation of results, and preparation of the manuscript draft. **Yangqiwen Luo** and **Cangning Zhang** were responsible for conducting experiments, collecting experimental data, and assisting with data interpretation. **Meng Ma** and **Genxi Zhang** contributed to experimental procedures, data collection, and verification of experimental results. **Jiying Liu** and **Liumei Sun** contributed to experimental materials, experimental implementation, and verification of experimental results. **Manman Shen** and **Liang Qu** supervised the overall study, provided scientific guidance, developed the research concept, secured research support, critically revised the manuscript, and were responsible for the overall integrity and quality control of the study.

## Disclosures

The authors declare that they have no conflicts of interest.

## Appendix Supplementary materials

Image, application 1

Image, application 2

## Footnotes

Supplementary material associated with this article can be found, in the online version, at doi:10.1016/j.psj.2026.107399.
