# Supplementary material for: GSK3β inhibits the differentiation of follicular granulosa cells by promoting lipid accumulation through autophagy in chickens
Source: Poult Sci. 2026 Jul 5;105(10):107399. doi: 10.1016/j.psj.2026.107399 (PMC13427471; doi:10.1016/j.psj.2026.107399)
Supplement: Supplementary file 1 [file mmc1.pdf]

Figure 1A

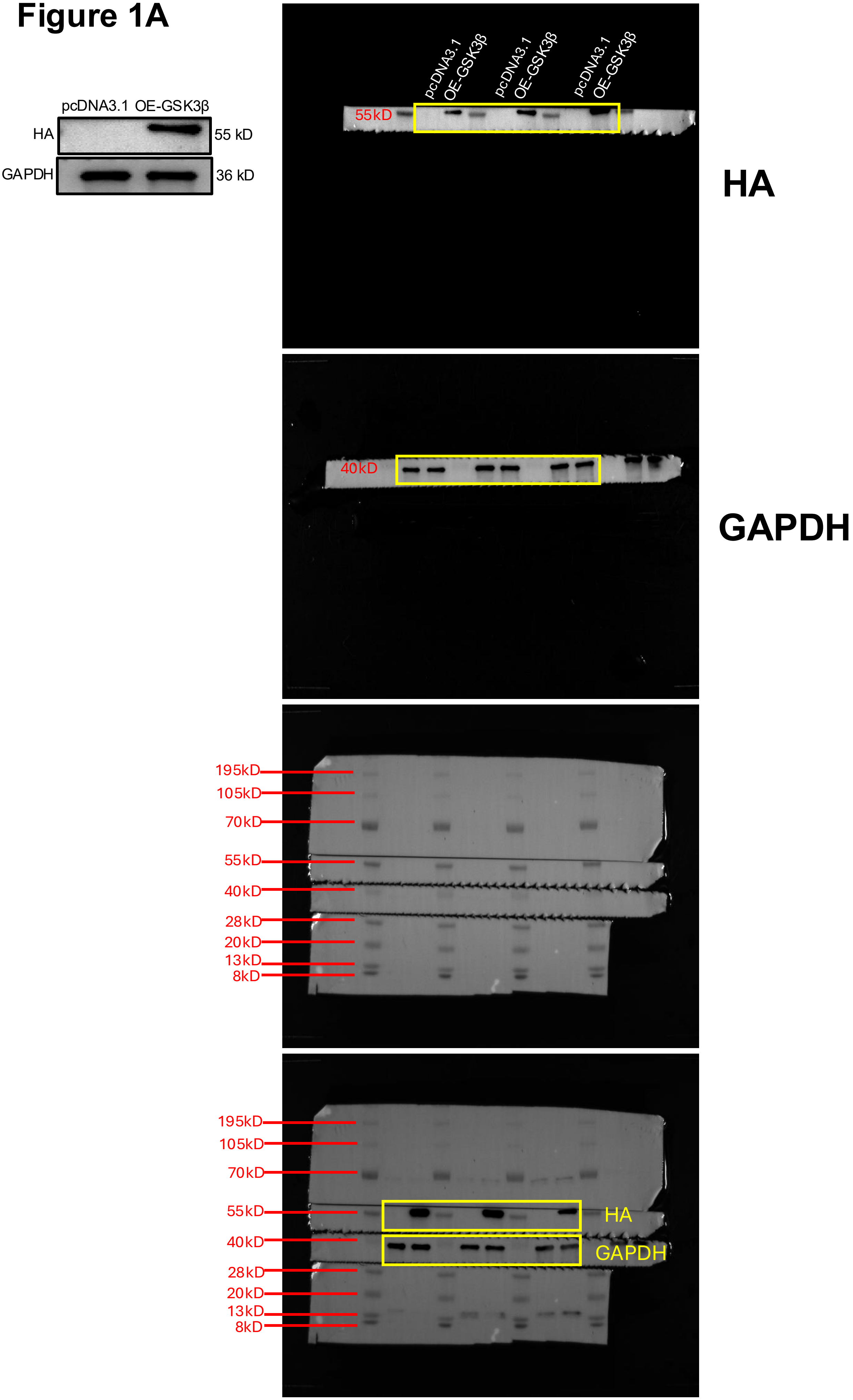
pcDNA3.1
OE-GSK3β
HA
55 kD
GAPDH
36 kD
pcDNA3.1
OE-GSK3β
pcDNA3.1
OE-GSK3β
pcDNA3.1
OE-GSK3β
55kD
HA
40kD
GAPDH
195kD
105kD
70kD
55kD
40kD
28kD
20kD
13kD
8kD
195kD
105kD
70kD
55kD
40kD
28kD
20kD
13kD
8kD
HA
GAPDH

Figure 2B

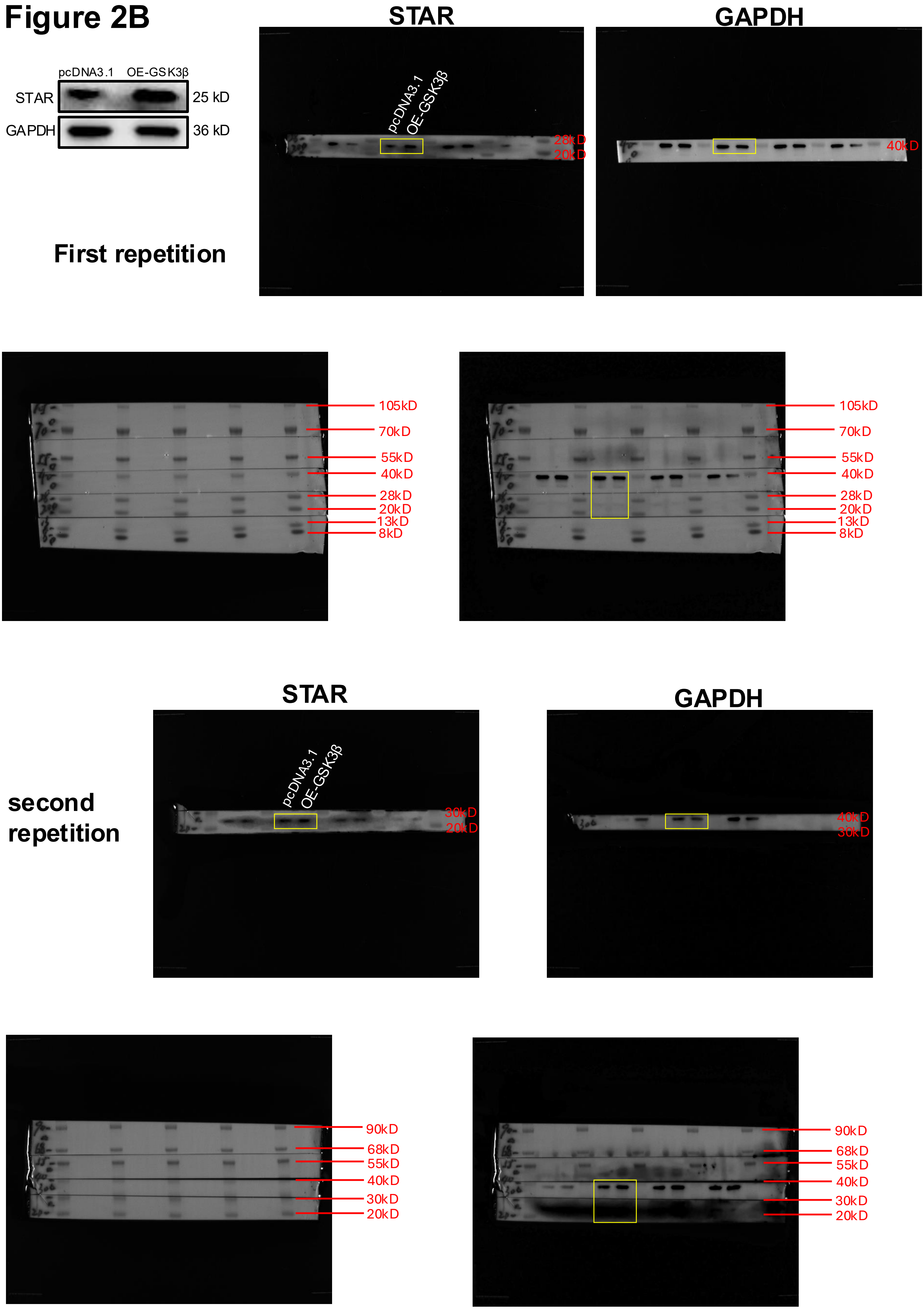
pcDNA3.1
OE-GSK3β
STAR
25 kD
GAPDH
36 kD
First repetition
STAR
GAPDH
pcDNA3.1
OE-GSK3β
28kD
20kD
40kD
105kD
70kD
55kD
40kD
28kD
20kD
13kD
8kD
105kD
70kD
55kD
40kD
28kD
20kD
13kD
8kD
second repetition
STAR
GAPDH
pcDNA3.1
OE-GSK3β
30kD
20kD
40kD
30kD
90kD
68kD
55kD
40kD
30kD
20kD
90kD
68kD
55kD
40kD
30kD
20kD

Figure 2B

Third repetition

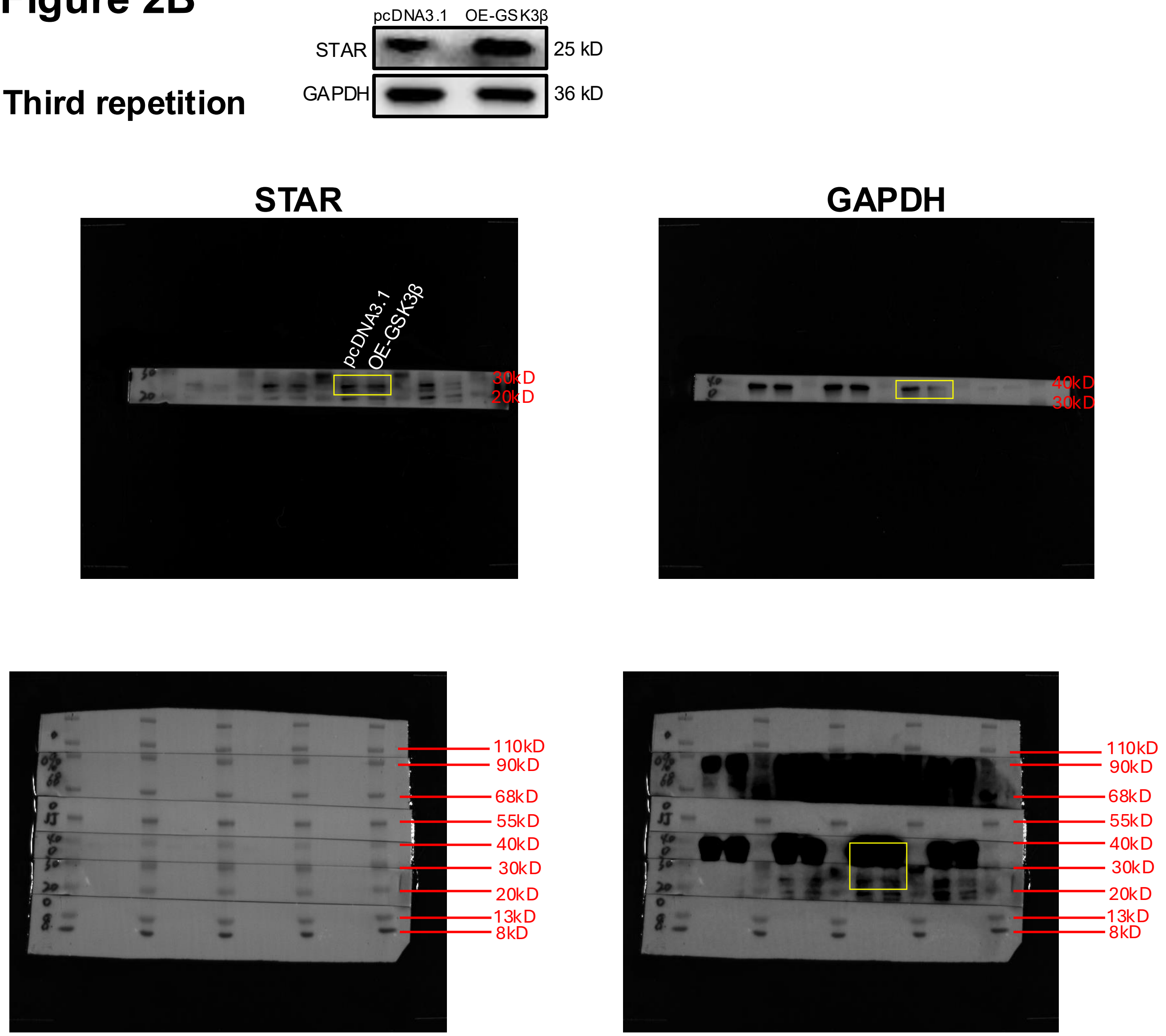

Figure 2B

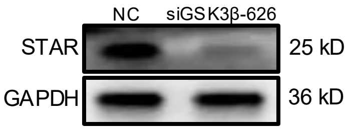

First repetition

STAR

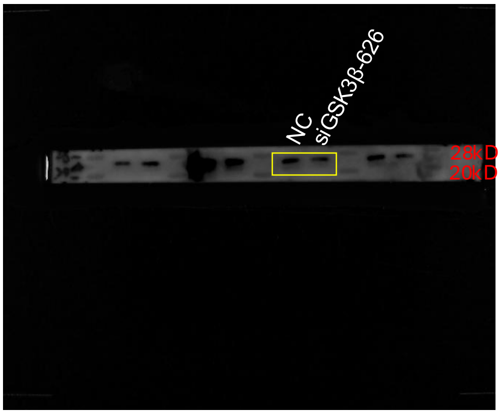

GAPDH

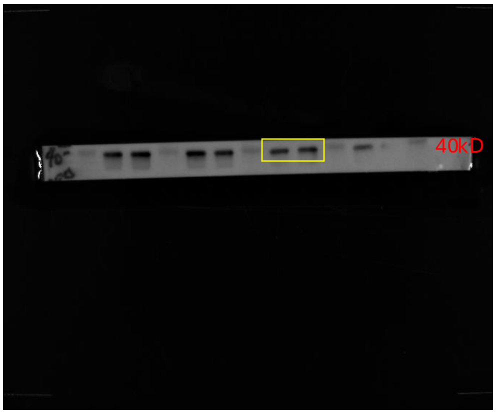

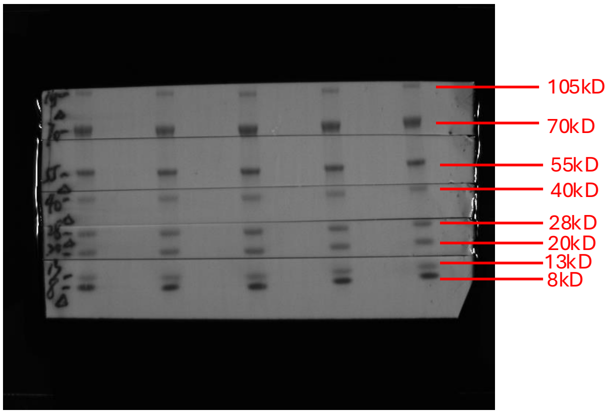

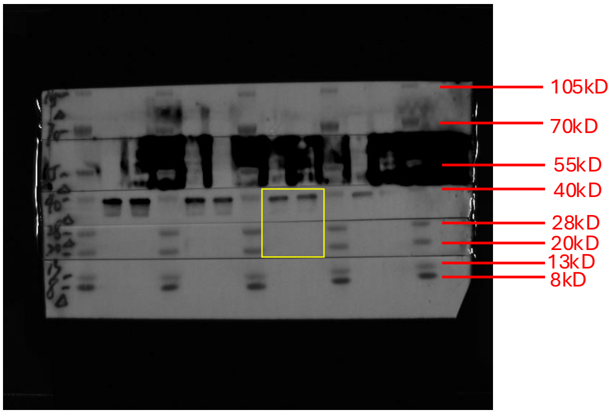

second and Third repetition

STAR

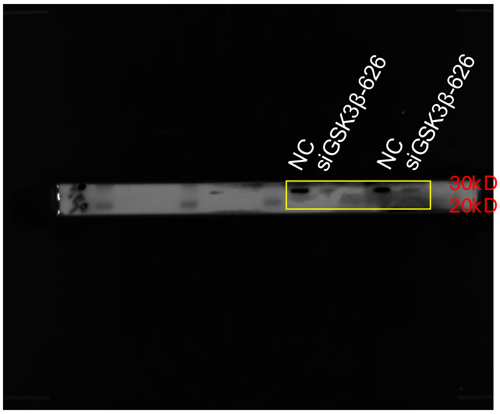

GAPDH

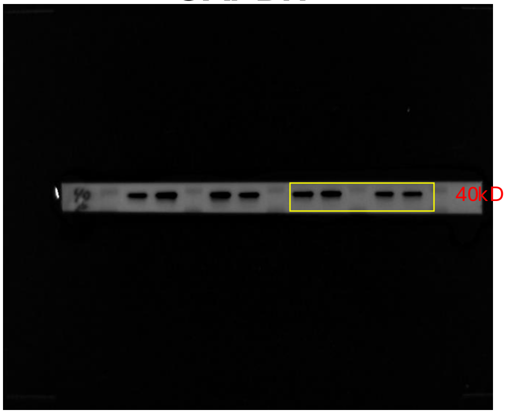

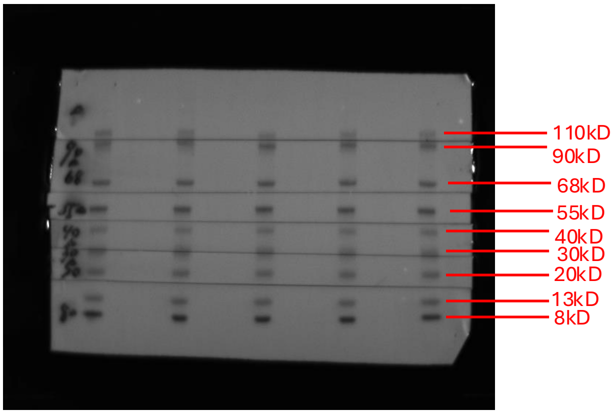

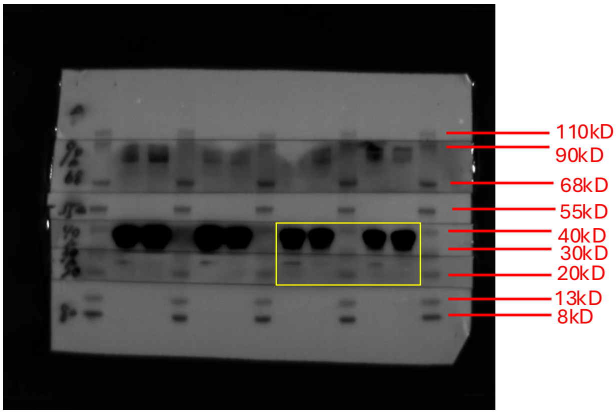

Figure 3B

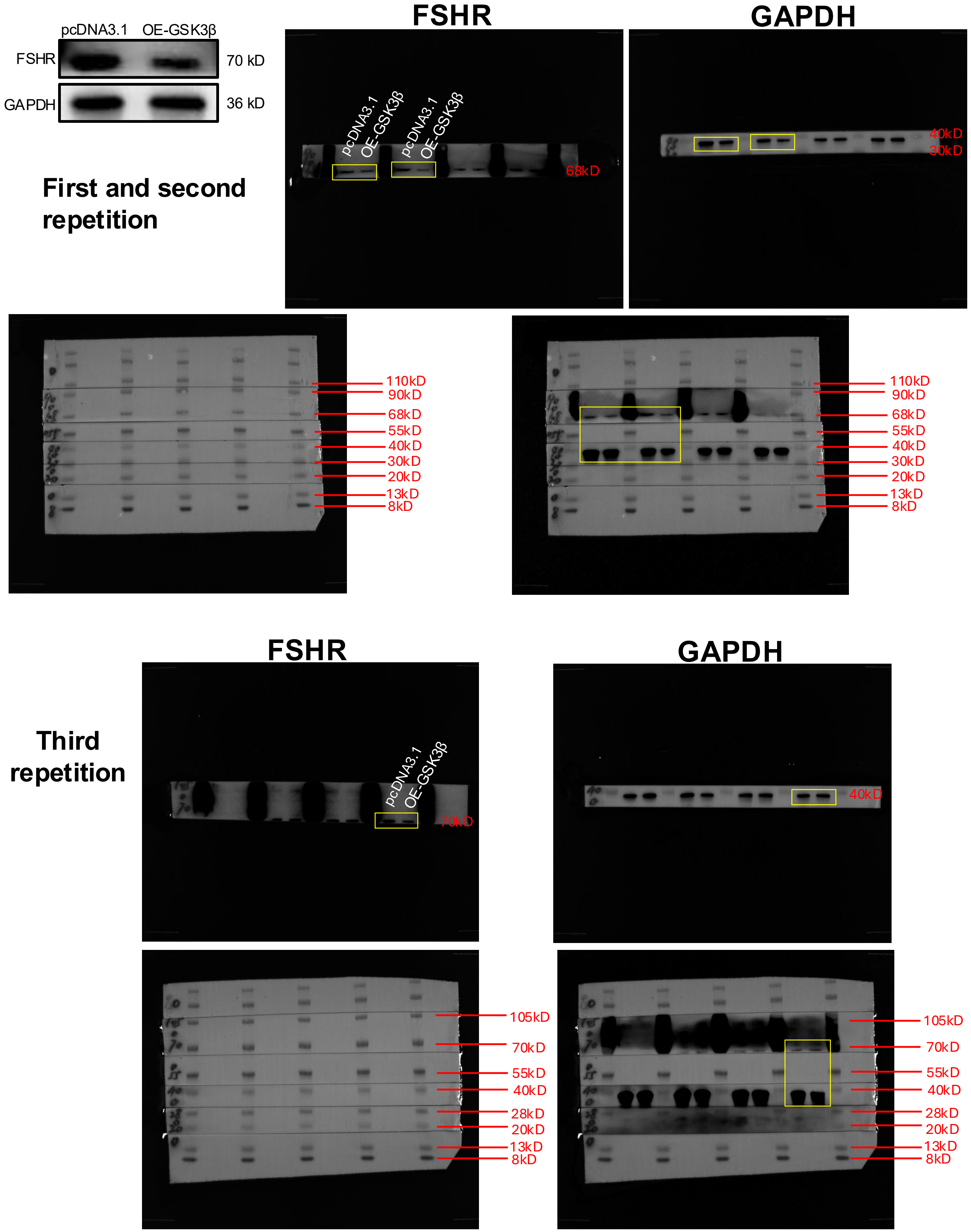

pcDNA3.1
OE-GSK3β
FSHR
GAPDH
70 kD
36 kD
First and second repetition
FSHR
GAPDH
68kD
40kD
30kD
110kD
90kD
68kD
55kD
40kD
30kD
20kD
13kD
8kD
Third repetition
70kD
40kD
105kD
70kD
55kD
40kD
28kD
20kD
13kD
8kD

Figure 3B

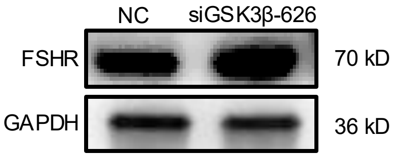

First and second repetition

FSHR

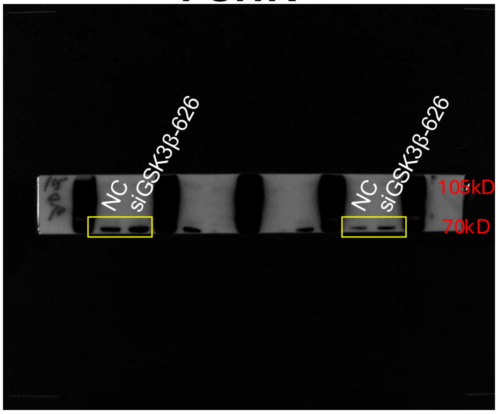

GAPDH

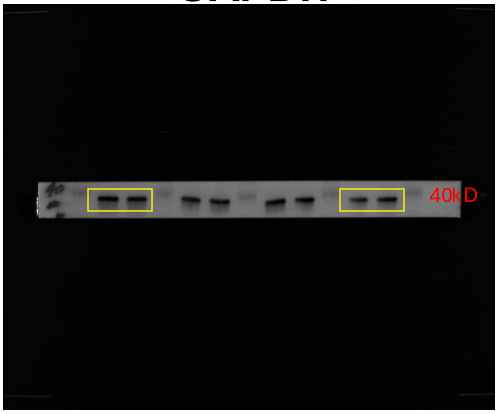

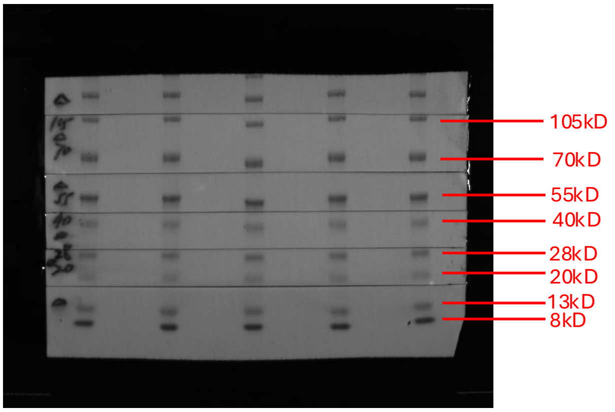

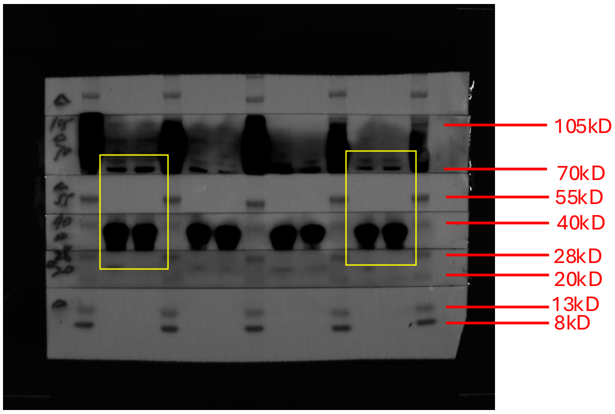

Third repetition

FSHR

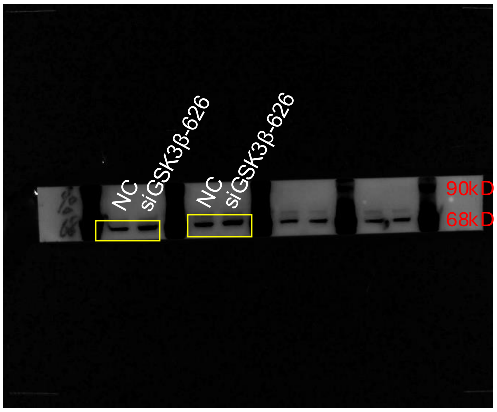

GAPDH

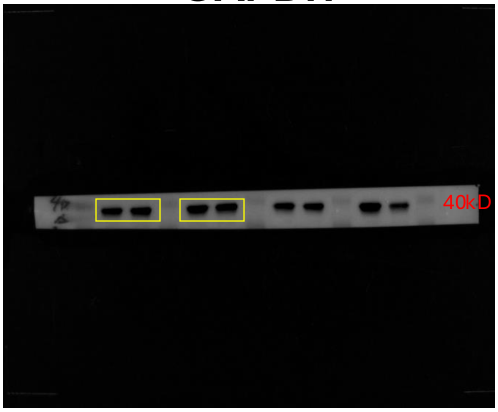

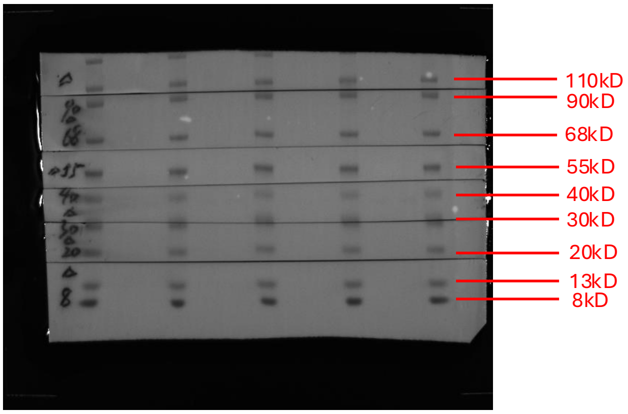

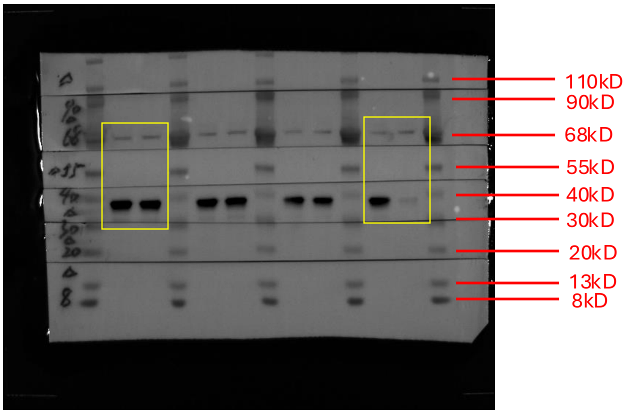

Figure 5A

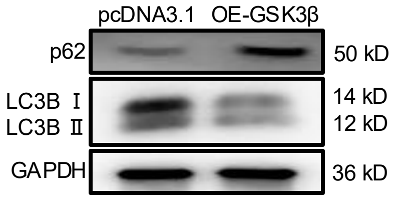

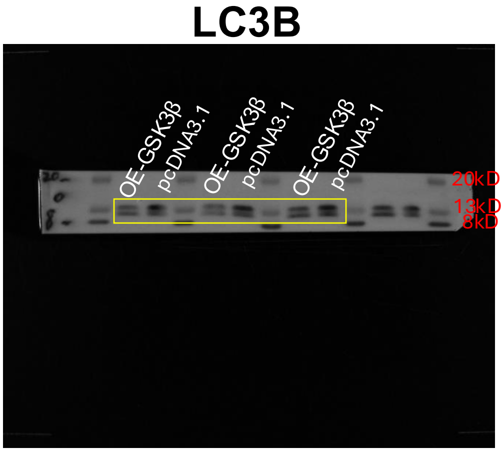

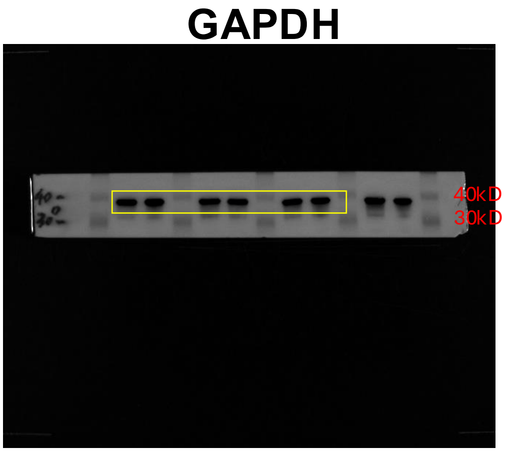

First, second, and third repetition

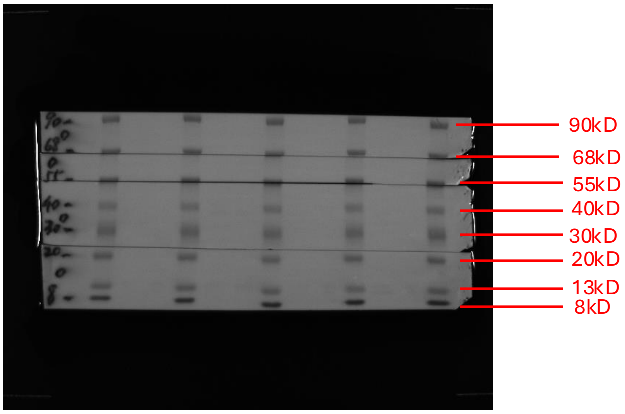

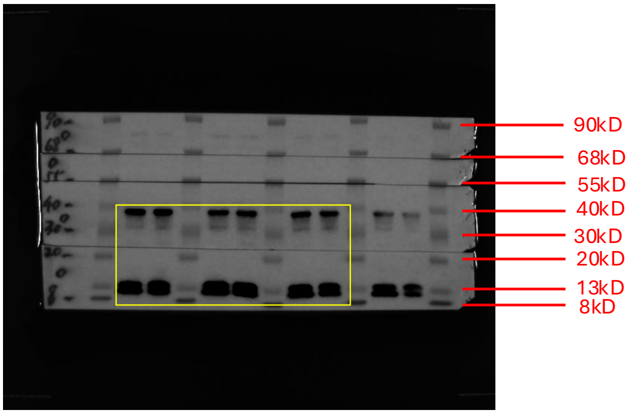

Fourth repetition

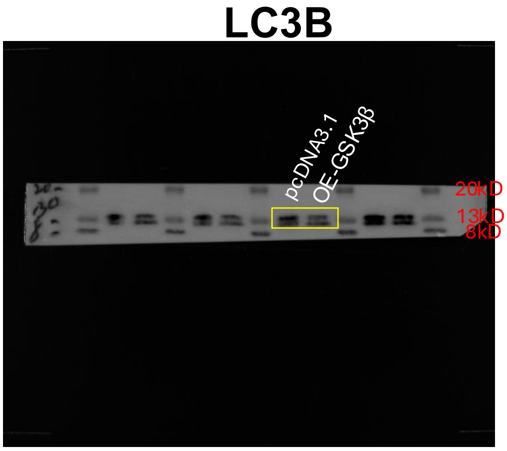

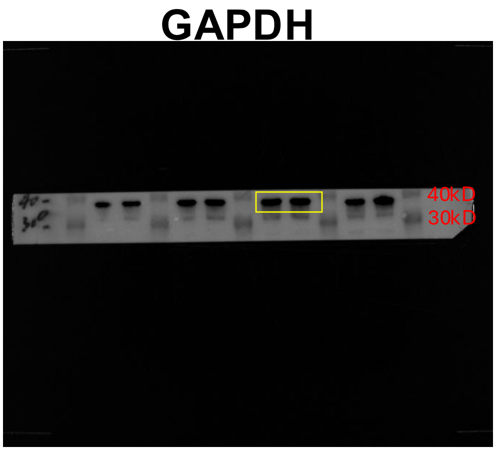

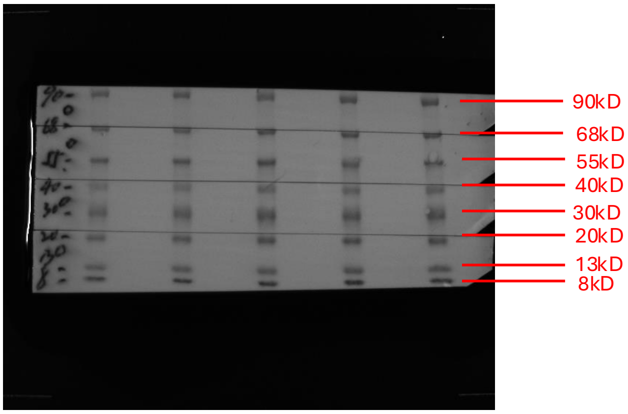

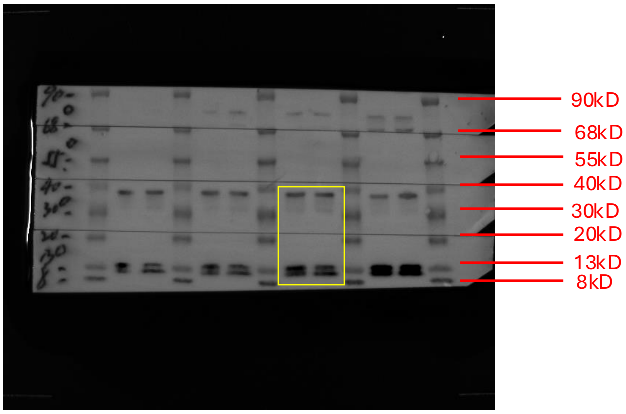

Figure 5A

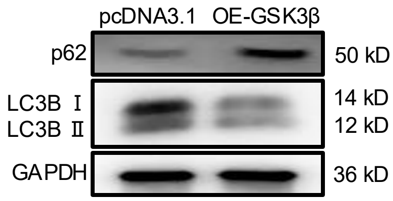

p62

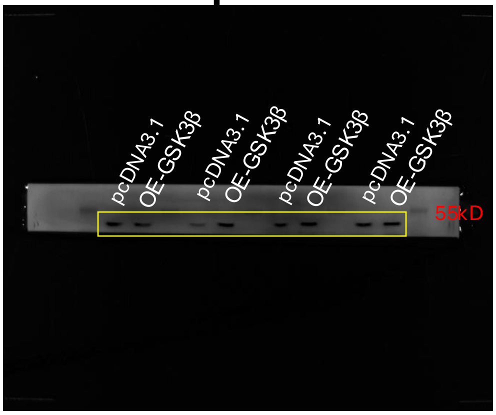

GAPDH

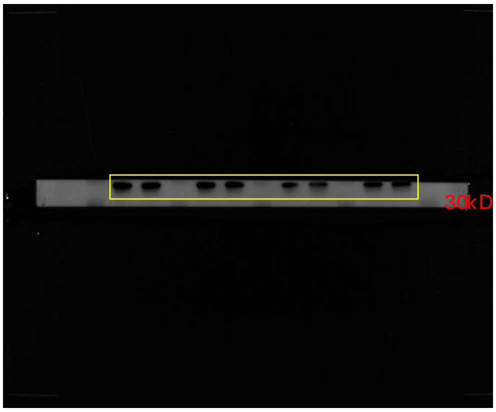

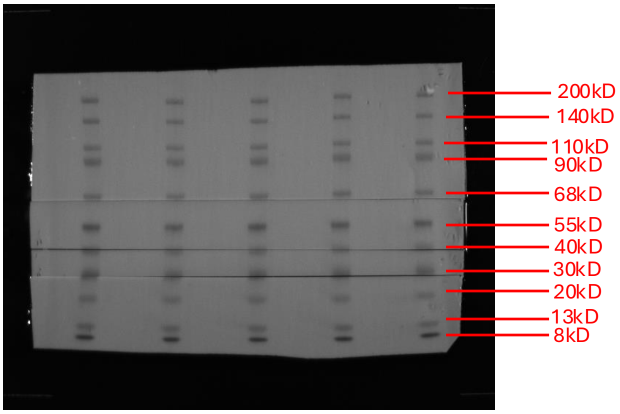

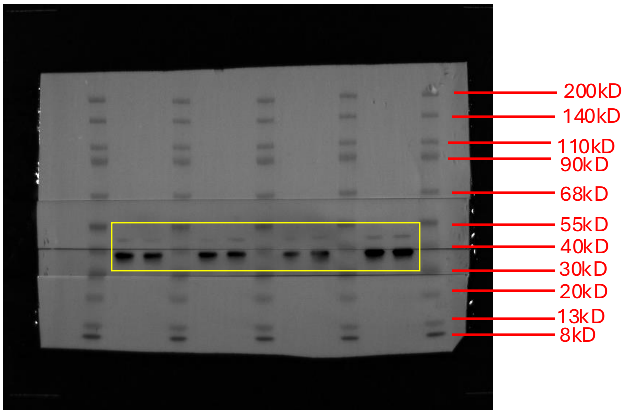

Figure 5A

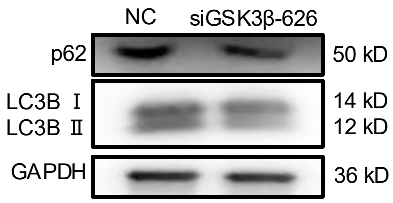

First and second repetition

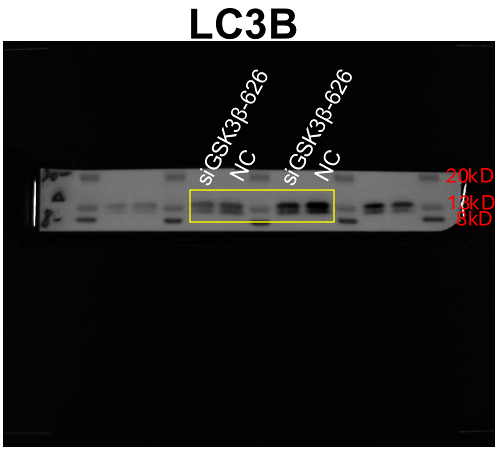

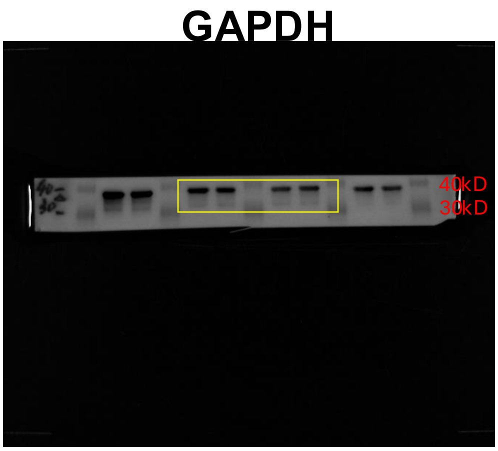

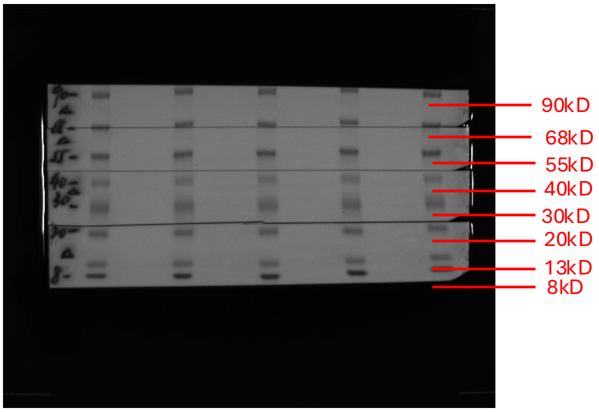

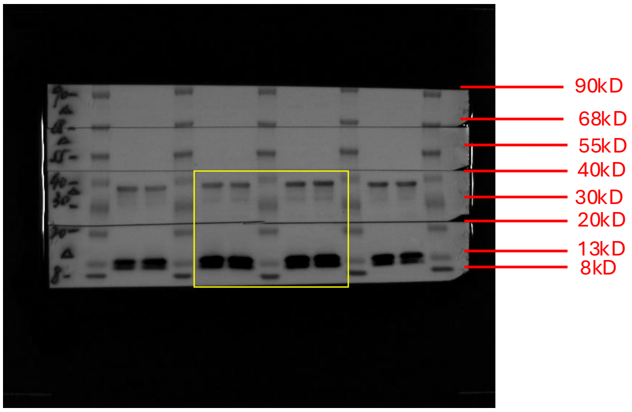

Third repetition

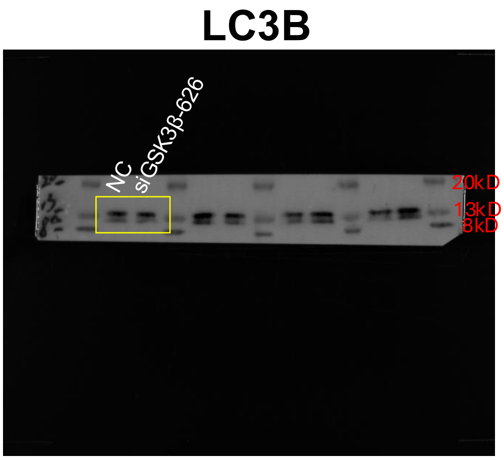

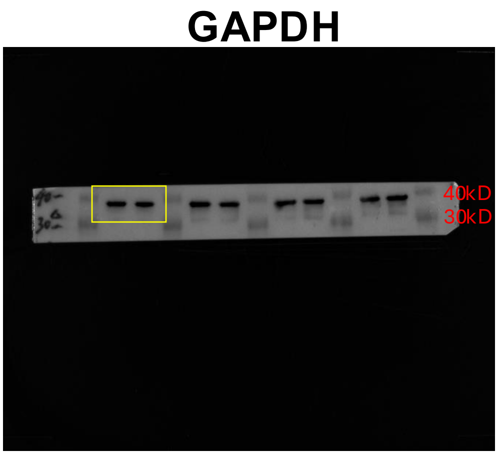

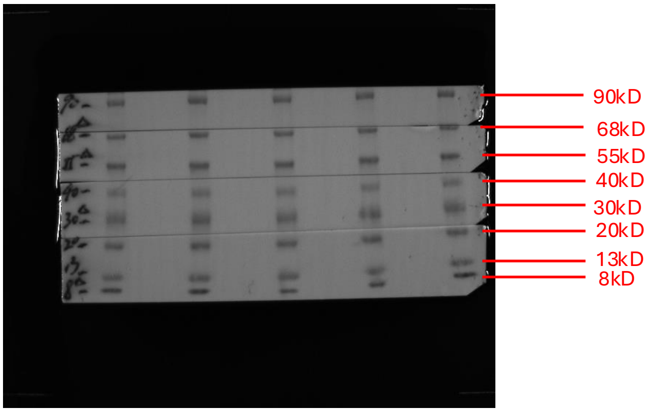

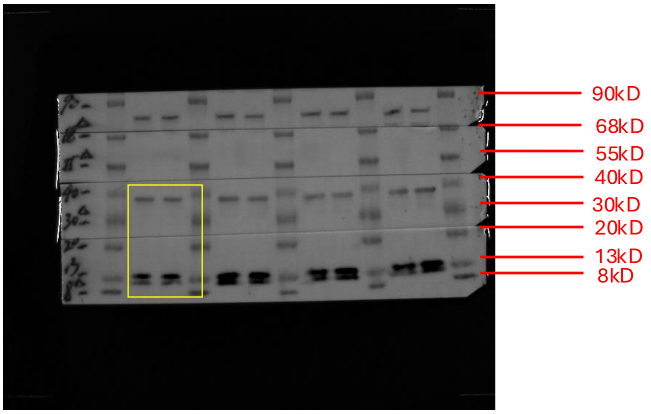

Figure 5A

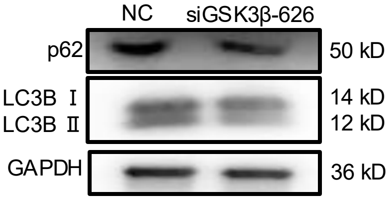

p62

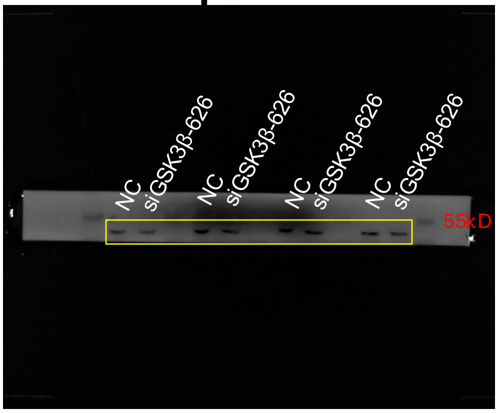

GAPDH

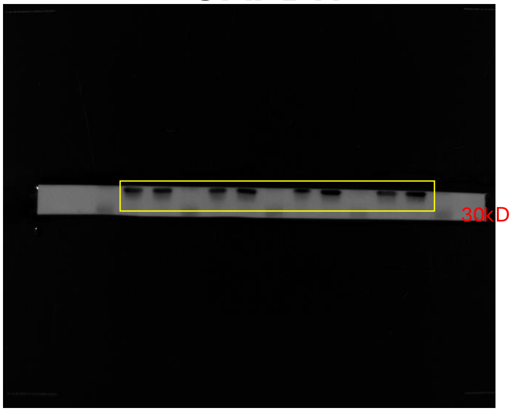

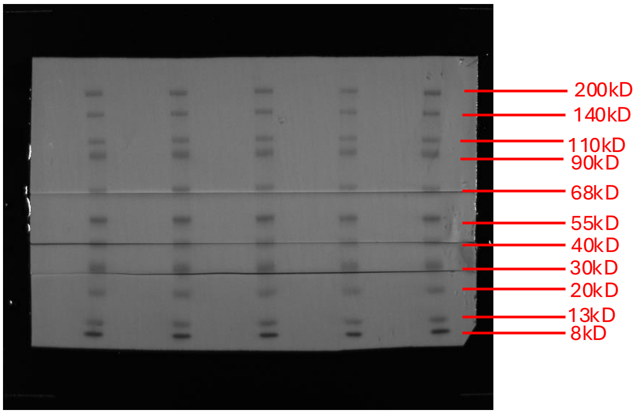

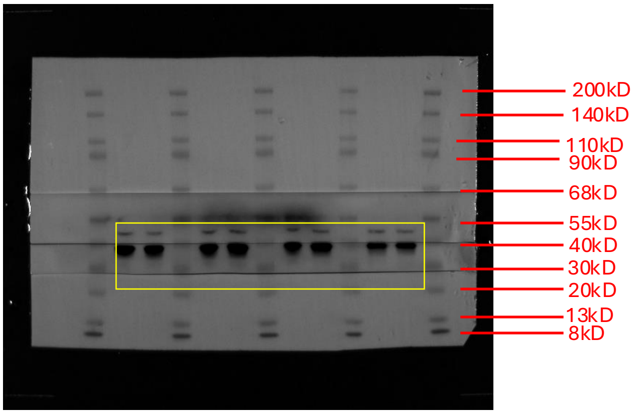

Figure 6A

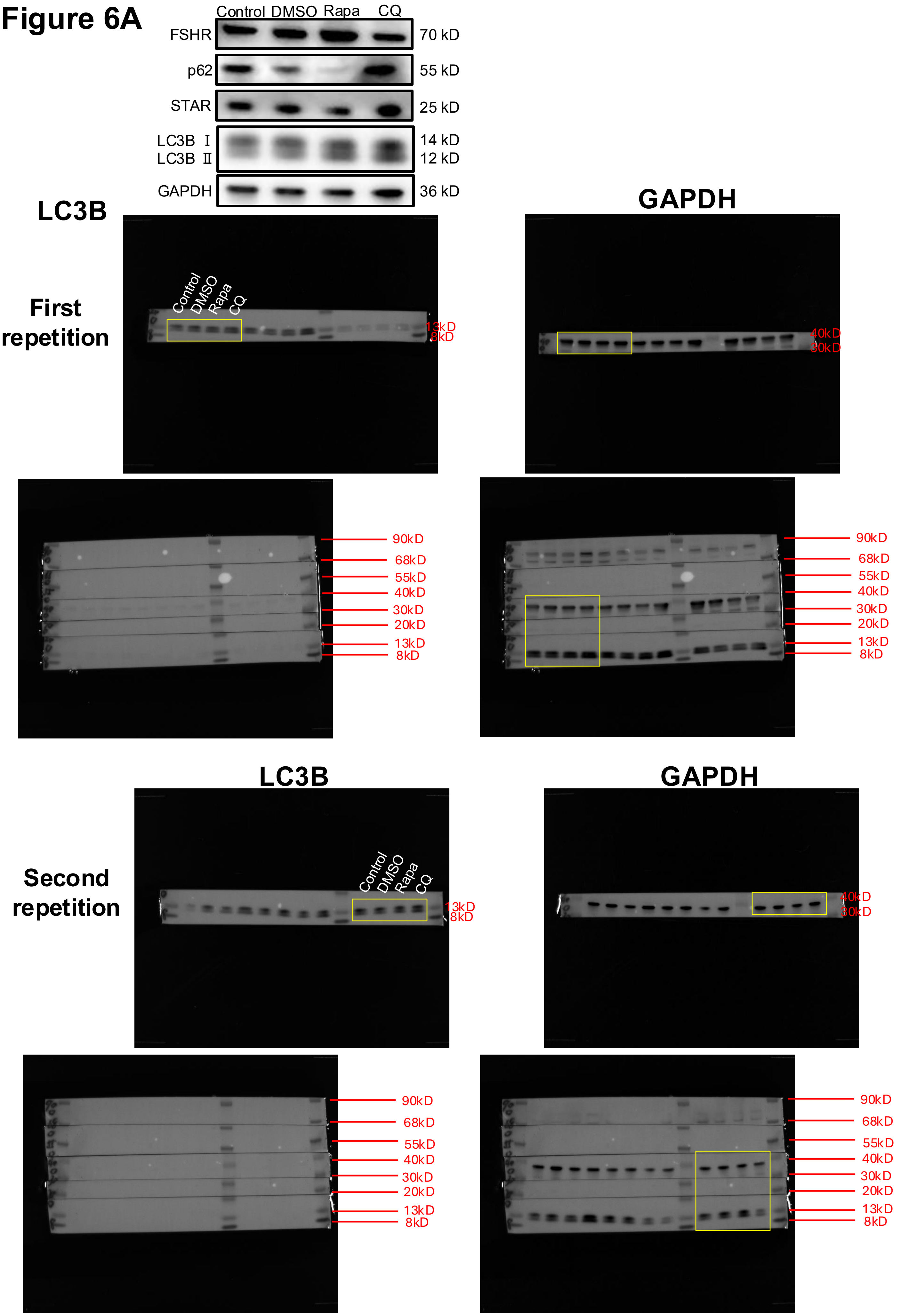

Control DMSO Rapa CQ
FSHR
70 kD
p62
55 kD
STAR
25 kD
LC3B Ⅰ
LC3B Ⅱ
14 kD
12 kD
GAPDH
36 kD
LC3B
GAPDH
First repetition
Control
DMSO
Rapa
CQ
13kD
8kD
40kD
30kD
90kD
68kD
55kD
40kD
30kD
20kD
13kD
8kD
LC3B
GAPDH
Second repetition
Control
DMSO
Rapa
CQ
13kD
8kD
40kD
30kD
90kD
68kD
55kD
40kD
30kD
20kD
13kD
8kD

Figure 6A

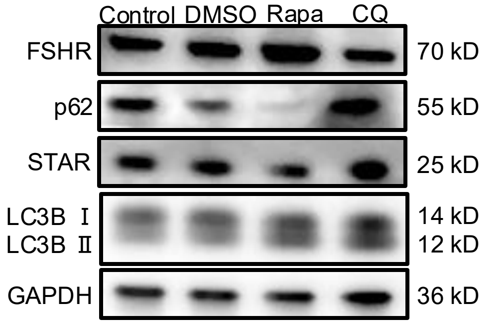

Third and fourth repetition

LC3B

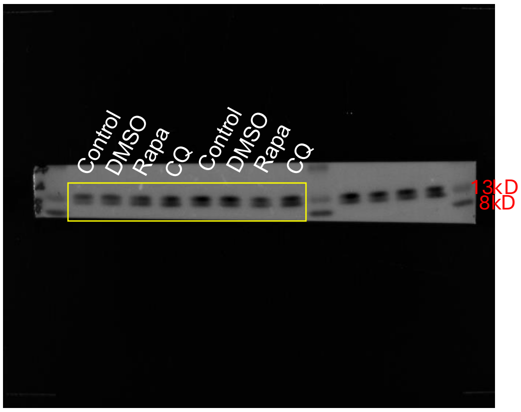

GAPDH

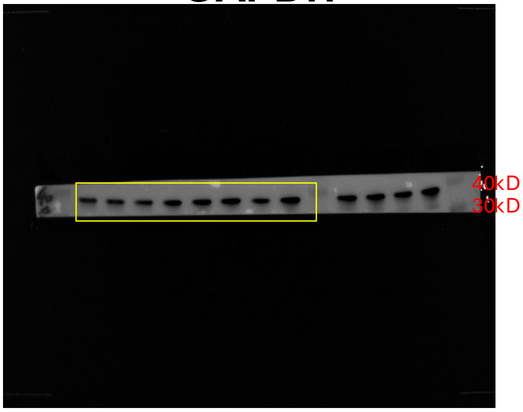

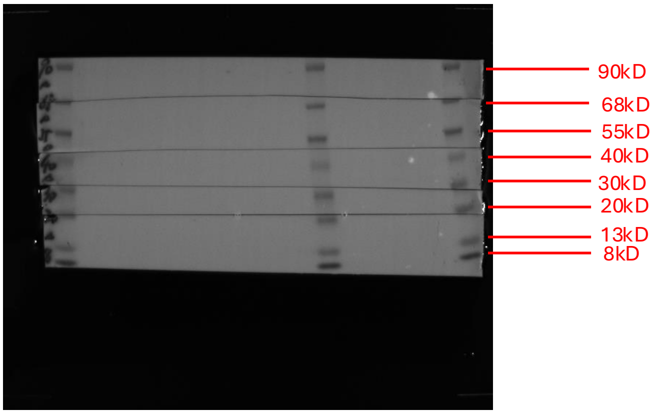

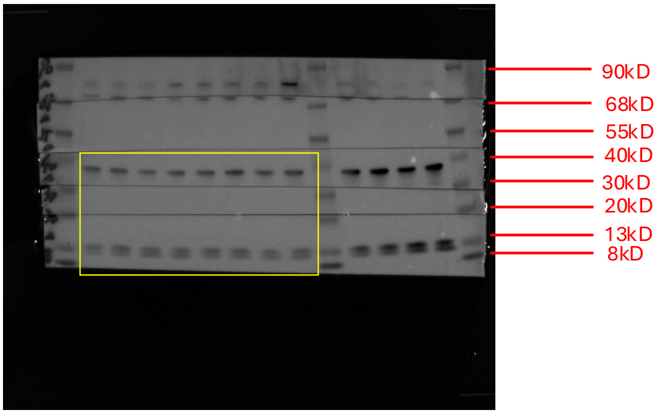

Figure 6A

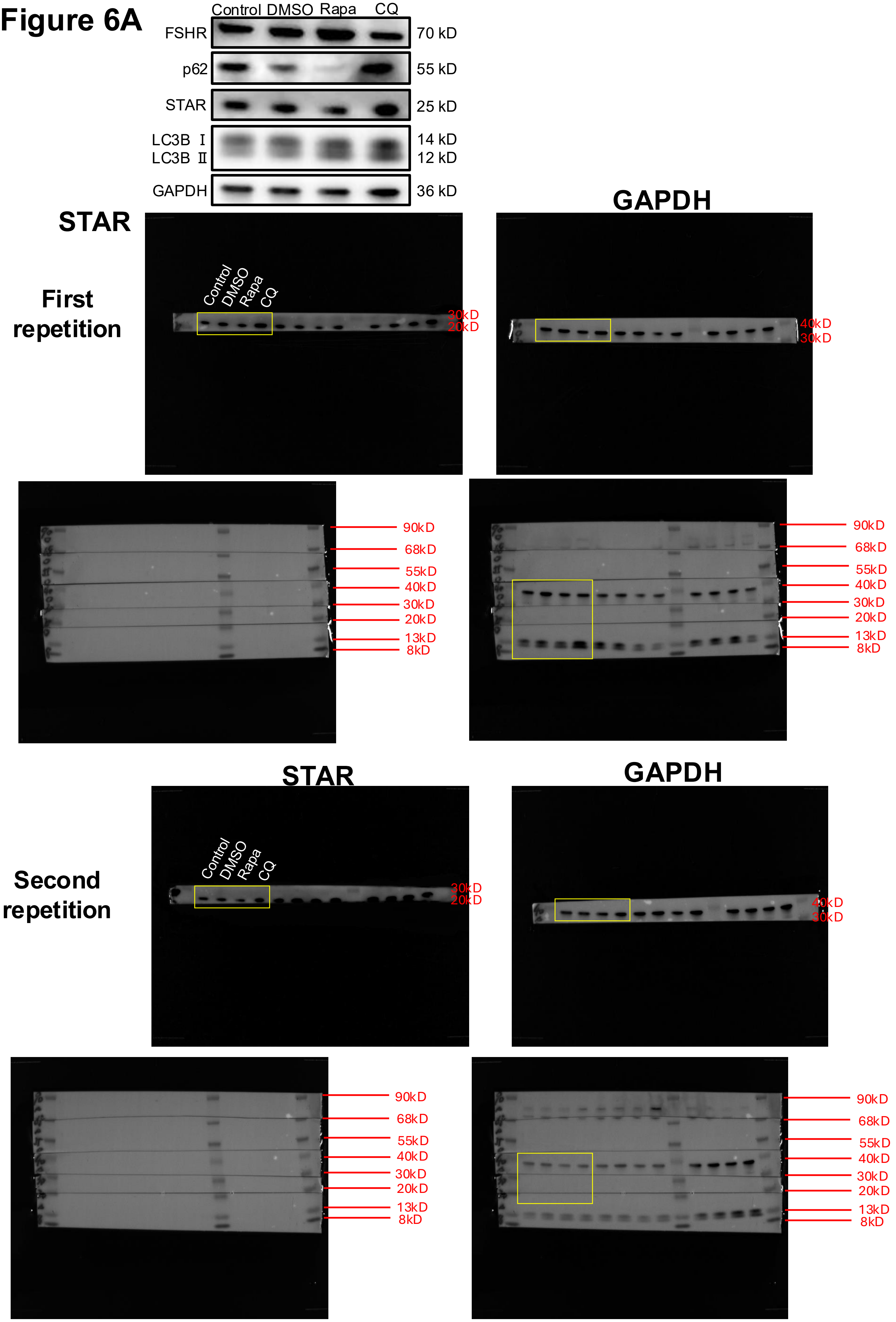

Control DMSO Rapa CQ
FSHR
70 kD
p62
55 kD
STAR
25 kD
LC3B Ⅰ
LC3B Ⅱ
14 kD
12 kD
GAPDH
36 kD
STAR
GAPDH
First repetition
Control
DMSO
Rapa
CQ
30kD
20kD
40kD
30kD
90kD
68kD
55kD
40kD
30kD
20kD
13kD
8kD
STAR
GAPDH
Second repetition
Control
DMSO
Rapa
CQ
30kD
20kD
40kD
30kD
90kD
68kD
55kD
40kD
30kD
20kD
13kD
8kD

Figure 6A

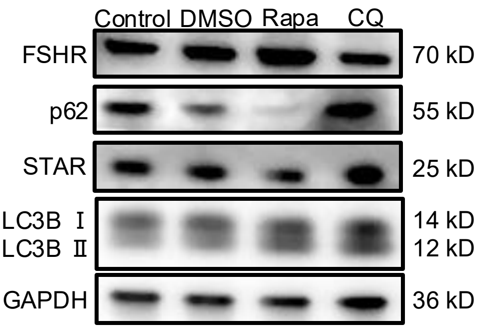

Third repetition

STAR

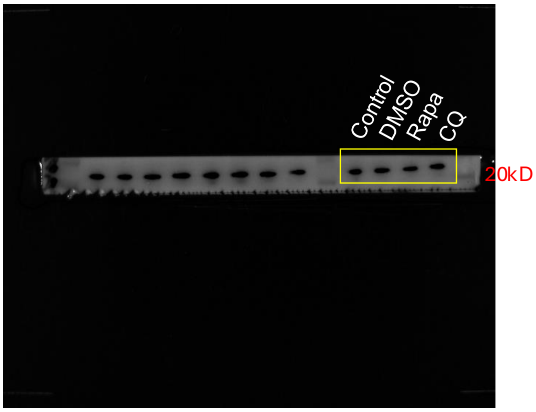

GAPDH

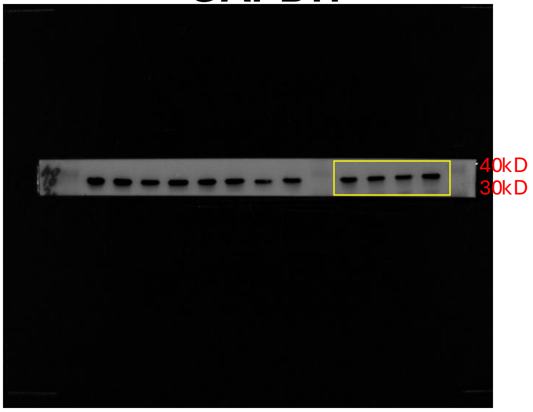

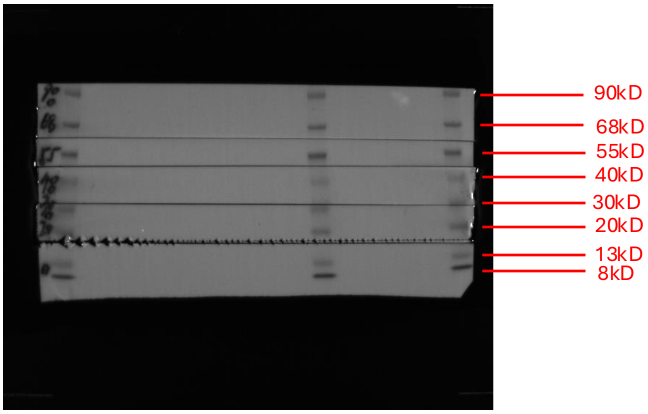

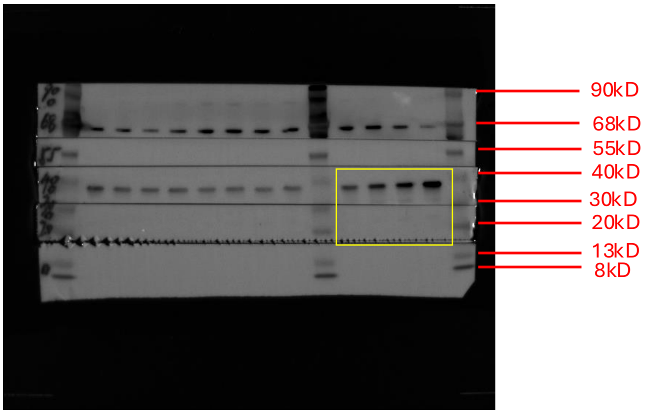

Figure 6A

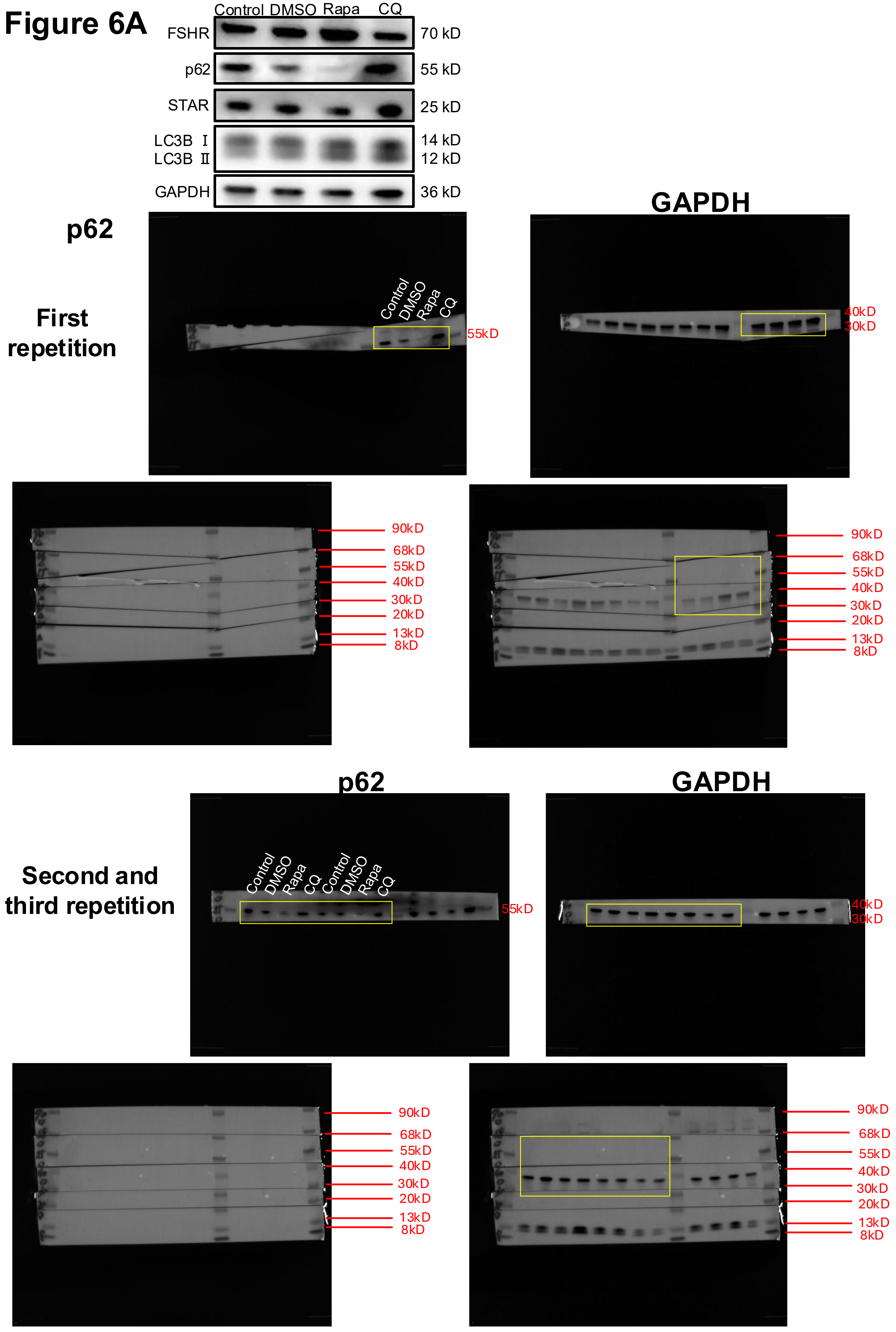

Control
DMSO
Rapa
CQ
FSHR
70 kD
p62
55 kD
STAR
25 kD
LC3B Ⅰ
LC3B Ⅱ
14 kD
12 kD
GAPDH
36 kD
p62
GAPDH
First repetition
Control
DMSO
Rapa
CQ
55kD
40kD
30kD
90kD
68kD
55kD
40kD
30kD
20kD
13kD
8kD
p62
GAPDH
Second and third repetition
Control
DMSO
Rapa
CQ
Control
DMSO
Rapa
CQ
55kD
40kD
30kD
90kD
68kD
55kD
40kD
30kD
20kD
13kD
8kD

Figure 6A

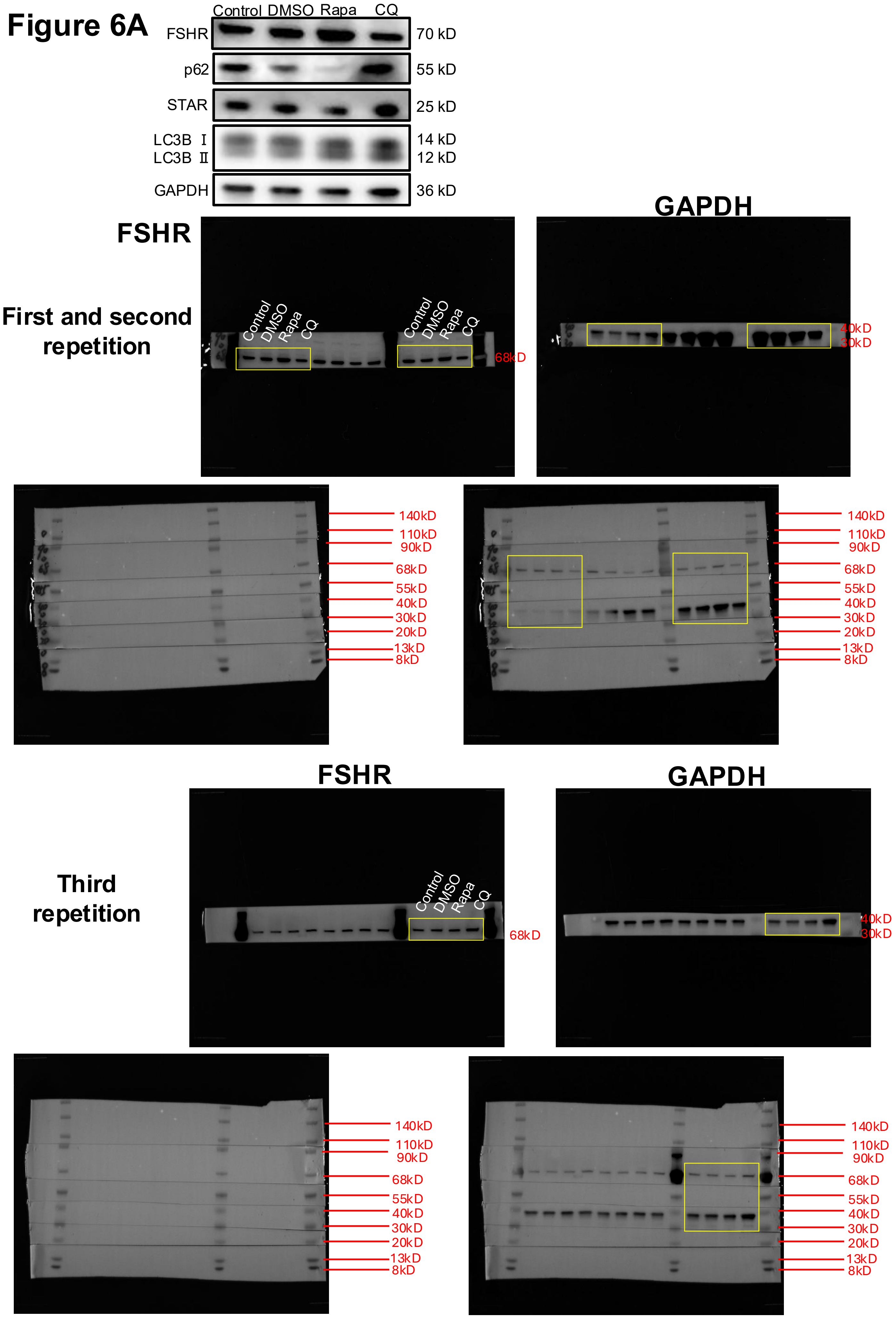
Control DMSO Rapa CQ
FSHR
70 kD
p62
55 kD
STAR
25 kD
LC3B I
LC3B II
14 kD
12 kD
GAPDH
36 kD
FSHR
GAPDH
First and second repetition
Control
DMSO
Rapa
CQ
68kD
40kD
30kD
140kD
110kD
90kD
68kD
55kD
40kD
30kD
20kD
13kD
8kD
FSHR
GAPDH
Third repetition
Control
DMSO
Rapa
CQ
68kD
40kD
30kD
140kD
110kD
90kD
68kD
55kD
40kD
30kD
20kD
13kD
8kD

Figure 8A

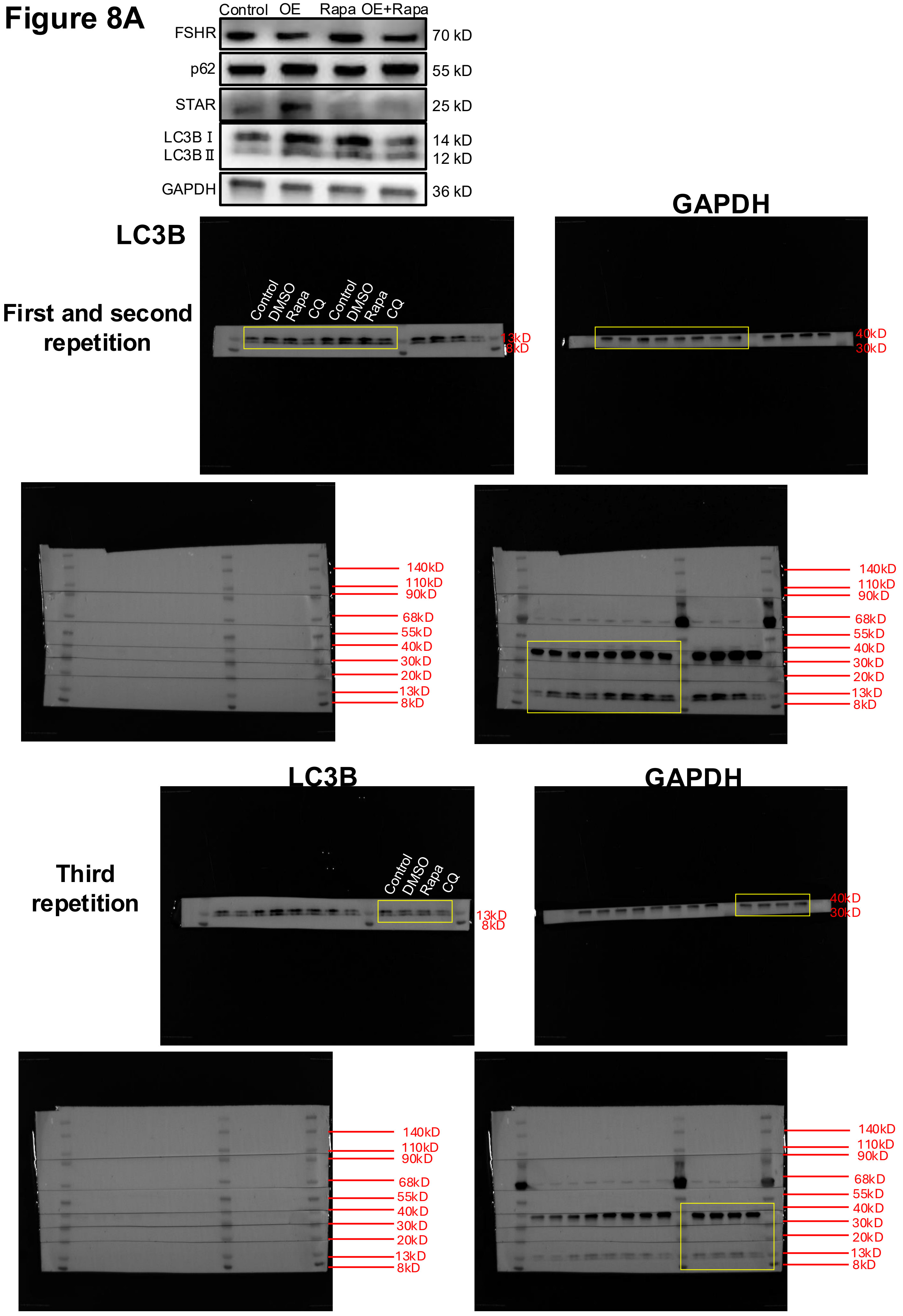

Control OE Rapa OE+Rapa
FSHR
70 kD
p62
55 kD
STAR
25 kD
LC3B I
LC3B II
14 kD
12 kD
GAPDH
36 kD
LC3B
GAPDH
First and second repetition
Control DMSO Rapa CQ Control DMSO Rapa CQ
13kD
8kD
40kD
30kD
140kD
110kD
90kD
68kD
55kD
40kD
30kD
20kD
13kD
8kD
LC3B
GAPDH
Third repetition
Control DMSO Rapa CQ
13kD
8kD
40kD
30kD
140kD
110kD
90kD
68kD
55kD
40kD
30kD
20kD
13kD
8kD

Figure 8A

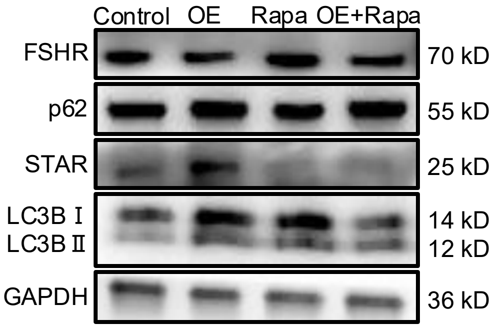

Fourth repetition

LC3B

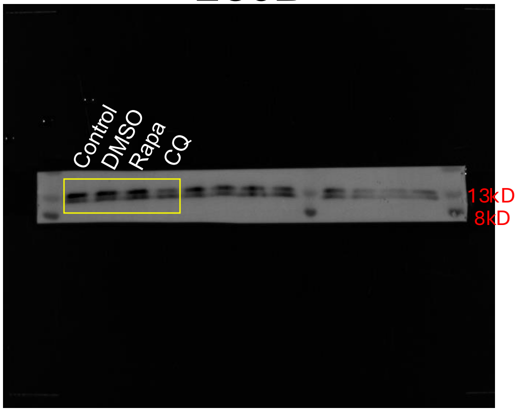

GAPDH

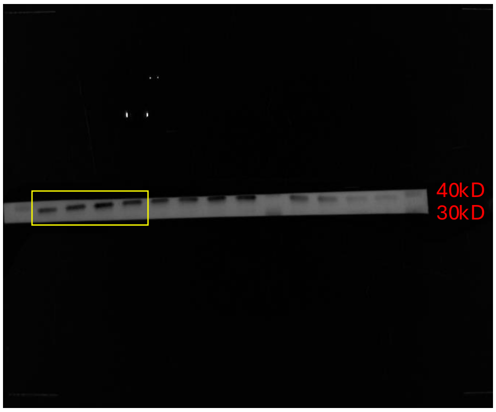

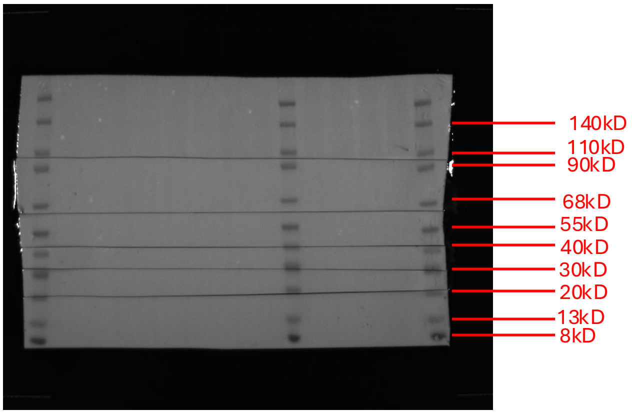

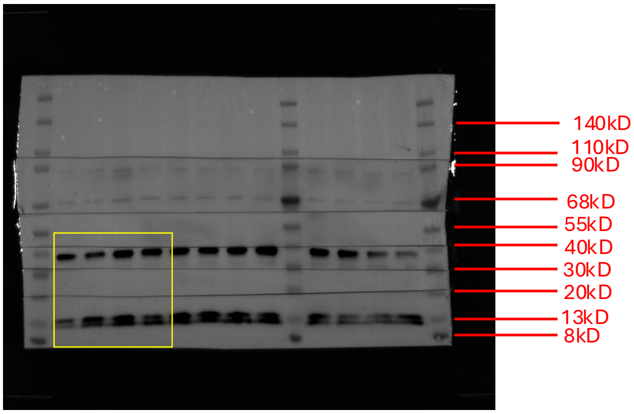

Figure 8A

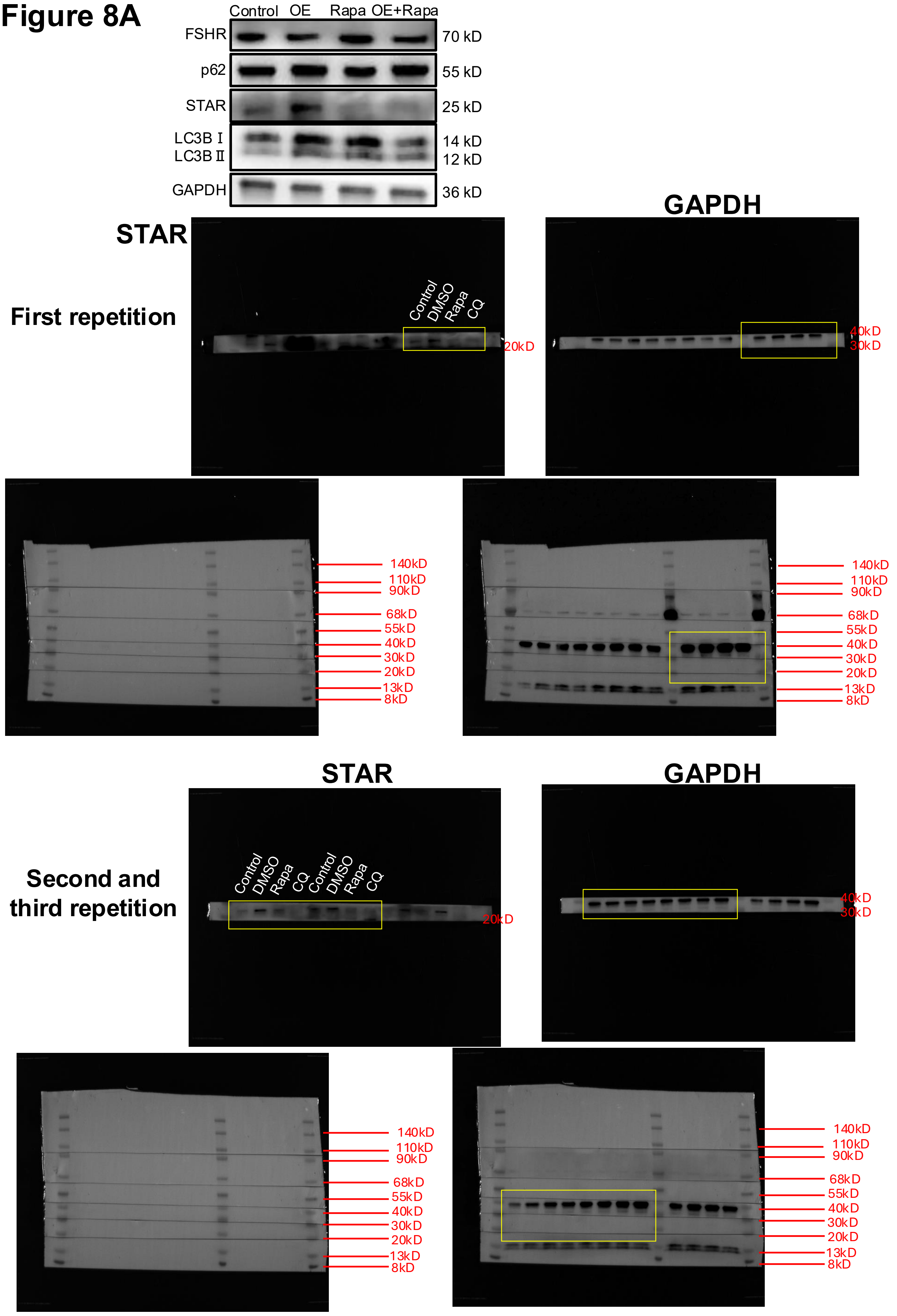

Control OE Rapa OE+Rapa
FSHR
70 kD
p62
55 kD
STAR
25 kD
LC3B Ⅰ
LC3B Ⅱ
14 kD
12 kD
GAPDH
36 kD
STAR
GAPDH
First repetition
Control
DMSO
Rapa
CQ
20kD
40kD
30kD
140kD
110kD
90kD
68kD
55kD
40kD
30kD
20kD
13kD
8kD
Second and third repetition
Control
DMSO
Rapa
CQ
Control
DMSO
Rapa
CQ
20kD
40kD
30kD

Figure 8A

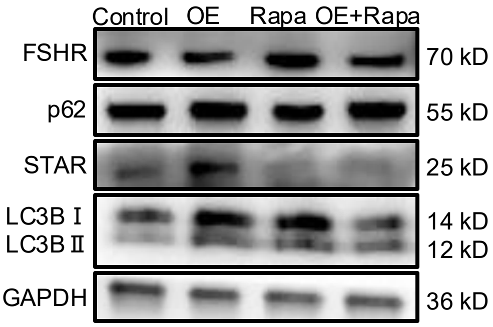

Fourth repetition

STAR

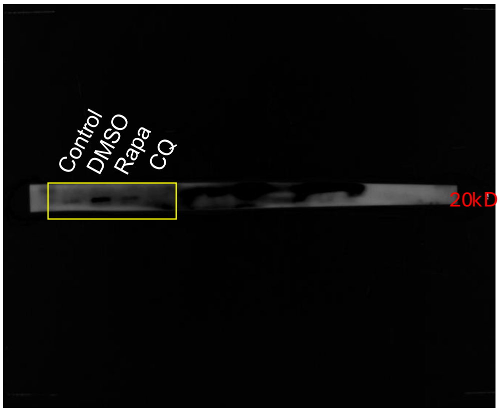

GAPDH

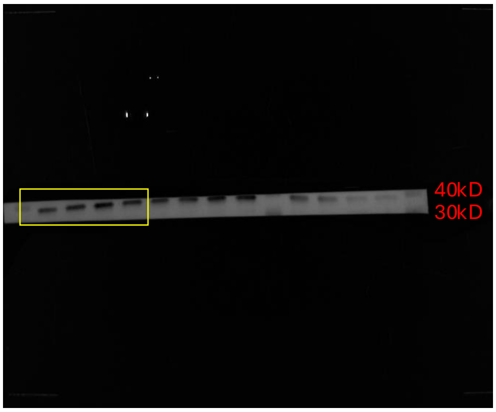

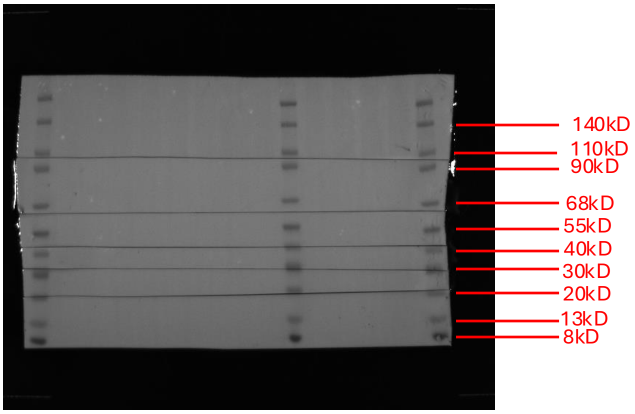

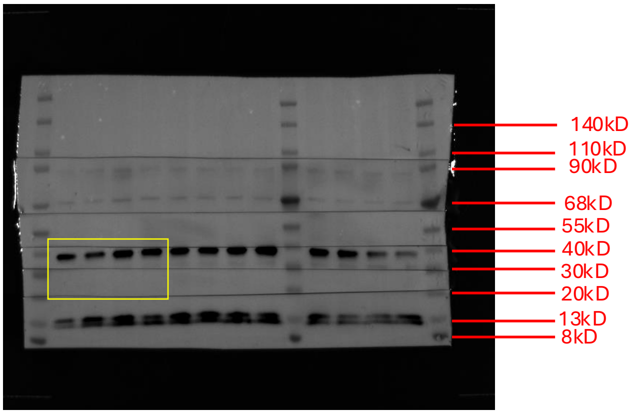

Figure 8A

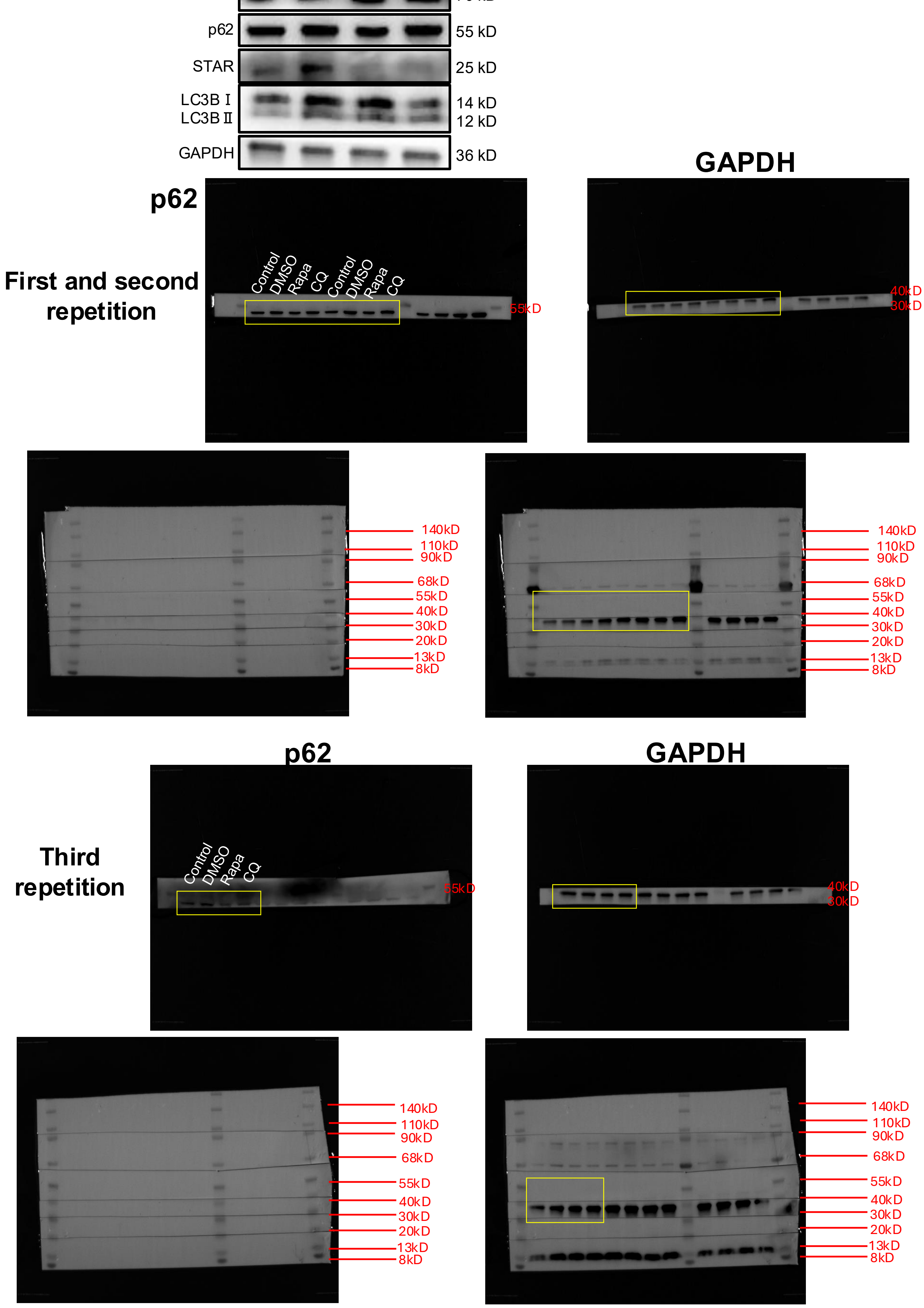
Control
OE
Rapa
OE+Rapa
FSHR
70 kD
p62
55 kD
STAR
25 kD
LC3B I
LC3B II
14 kD
12 kD
GAPDH
36 kD
p62
GAPDH
First and second repetition
Control
DMSO
Rapa
CQ
Control
DMSO
Rapa
CQ
55kD
40kD
30kD
140kD
110kD
90kD
68kD
55kD
40kD
30kD
20kD
13kD
8kD
p62
GAPDH
Third repetition
Control
DMSO
Rapa
CQ
55kD
40kD
30kD
140kD
110kD
90kD
68kD
55kD
40kD
30kD
20kD
13kD
8kD

Figure 8A

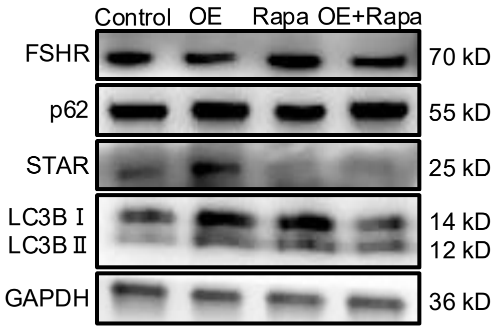

Fourth repetition

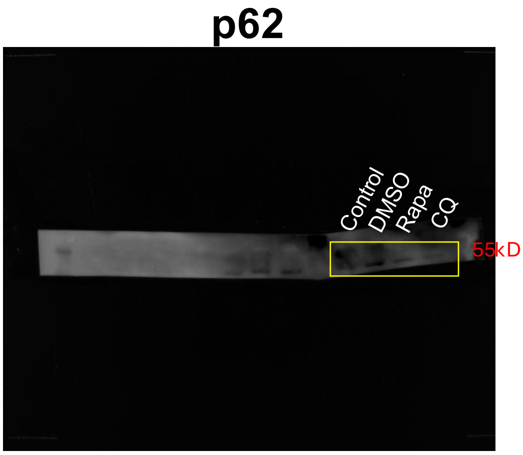

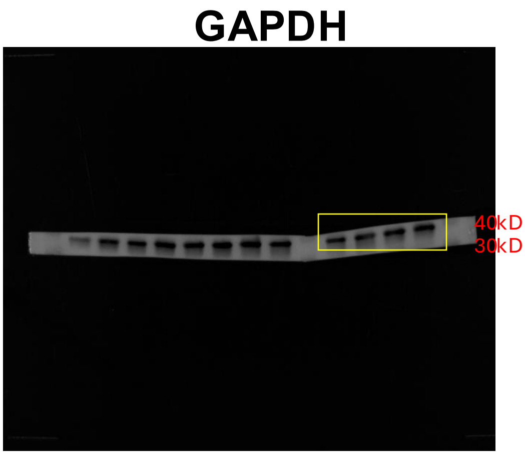

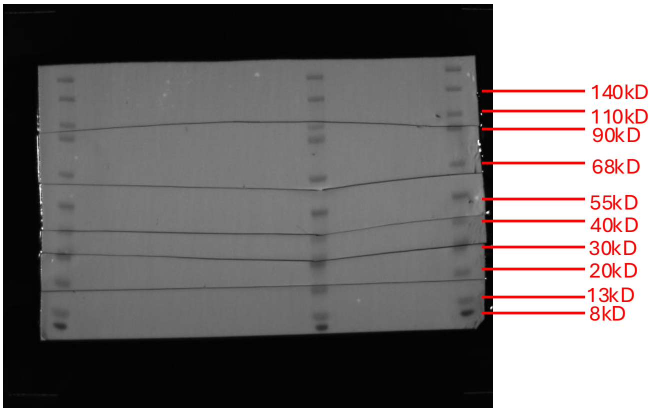

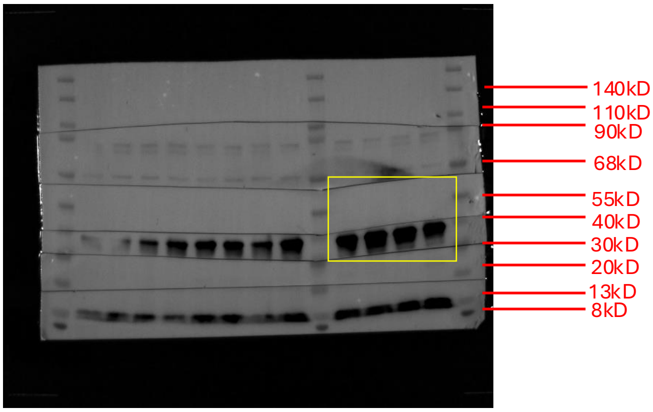

Figure 8A

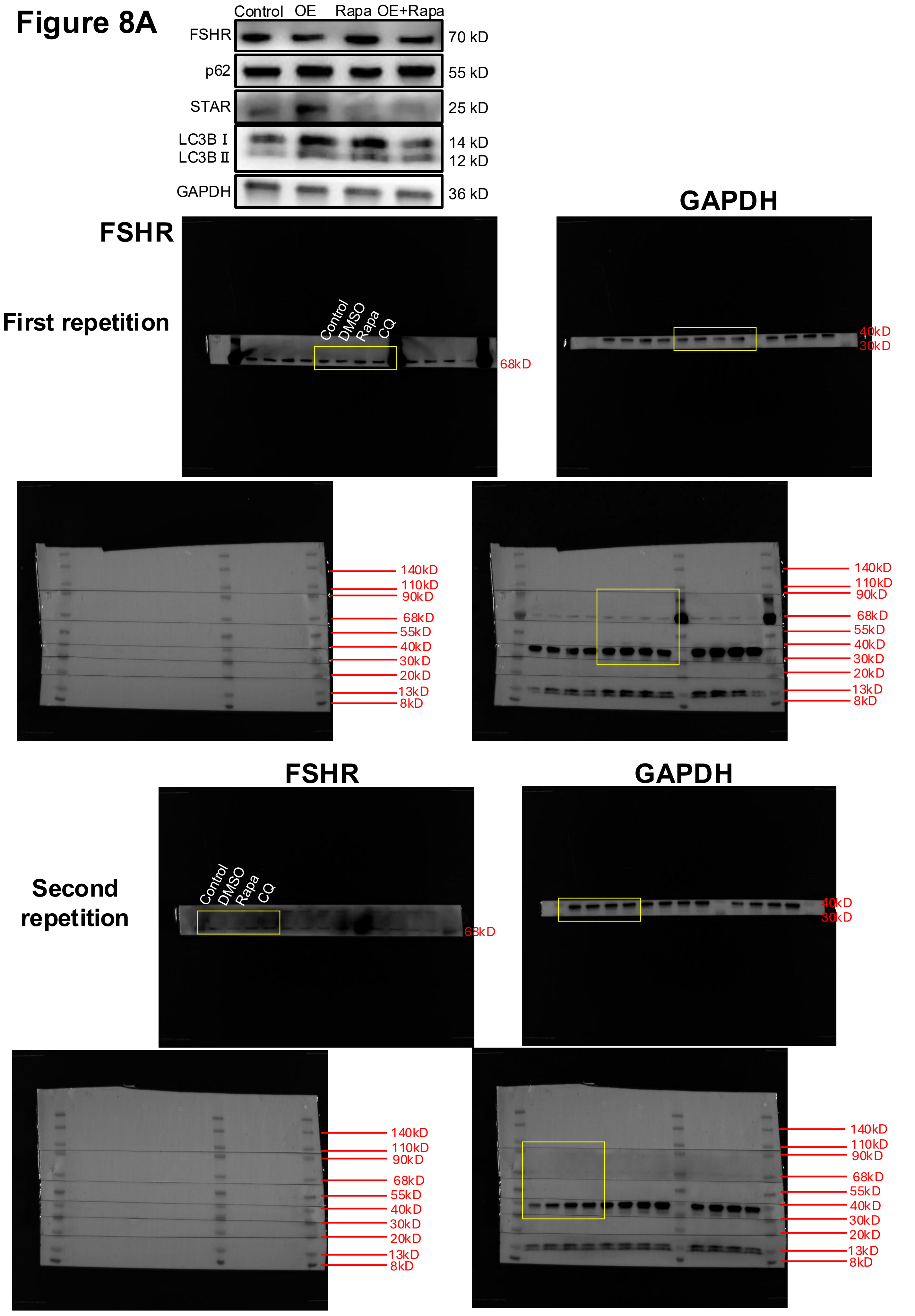

Control
OE
Rapa
OE+Rapa
FSHR
p62
STAR
LC3B I
LC3B II
GAPDH
70 kD
55 kD
25 kD
14 kD
12 kD
36 kD
FSHR
GAPDH
First repetition
Control
DMSO
Rapa
CQ
68kD
40kD
30kD
140kD
110kD
90kD
68kD
55kD
40kD
30kD
20kD
13kD
8kD
FSHR
GAPDH
Second repetition
Control
DMSO
Rapa
CQ
68kD
40kD
30kD
140kD
110kD
90kD
68kD
55kD
40kD
30kD
20kD
13kD
8kD

Figure 8A

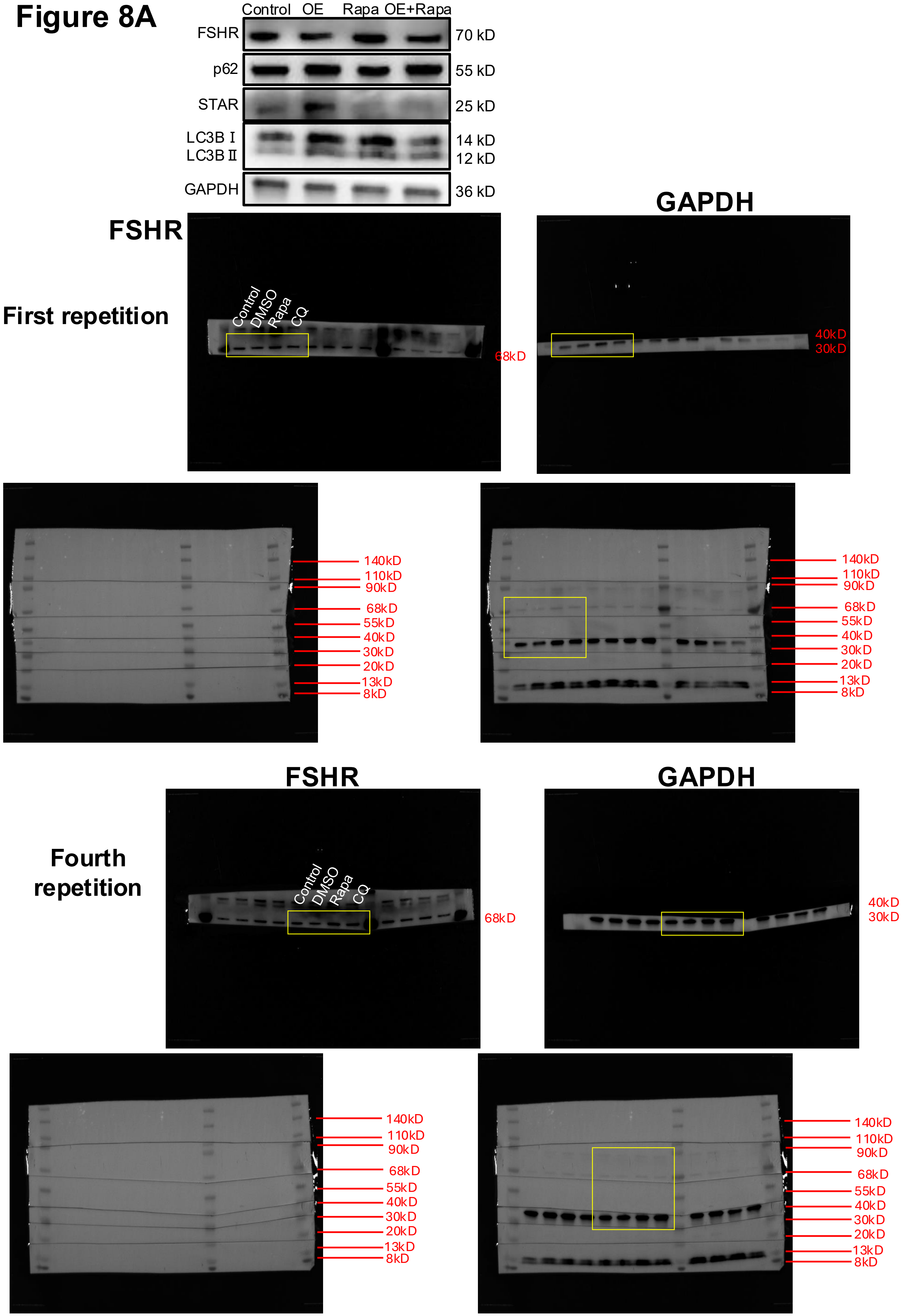
Control
OE
Rapa
OE+Rapa
FSHR
70 kD
p62
55 kD
STAR
25 kD
LC3B I
LC3B II
14 kD
12 kD
GAPDH
36 kD
FSHR
First repetition
Control
DMSO
Rapa
CQ
68kD
GAPDH
40kD
30kD
140kD
110kD
90kD
68kD
55kD
40kD
30kD
20kD
13kD
8kD
140kD
110kD
90kD
68kD
55kD
40kD
30kD
20kD
13kD
8kD
FSHR
GAPDH
Fourth repetition
Control
DMSO
Rapa
CQ
68kD
40kD
30kD
140kD
110kD
90kD
68kD
55kD
40kD
30kD
20kD
13kD
8kD
140kD
110kD
90kD
68kD
55kD
40kD
30kD
20kD
13kD
8kD
