# Supplementary material for: GSK3β inhibits the differentiation of follicular granulosa cells by promoting lipid accumulation through autophagy in chickens
Source: Poult Sci. 2026 Jul 5;105(10):107399. doi: 10.1016/j.psj.2026.107399 (PMC13427471; doi:10.1016/j.psj.2026.107399)

#### **Supplementary material**

**Figure. S1** A volcano plot illustrates the differentially expressed genes (DEGs) following treatment with pcDNA-N-HA-GSK3β in granulosa cells. (Red spots represent DEGs for upregulated, yellow pins for downregulated).


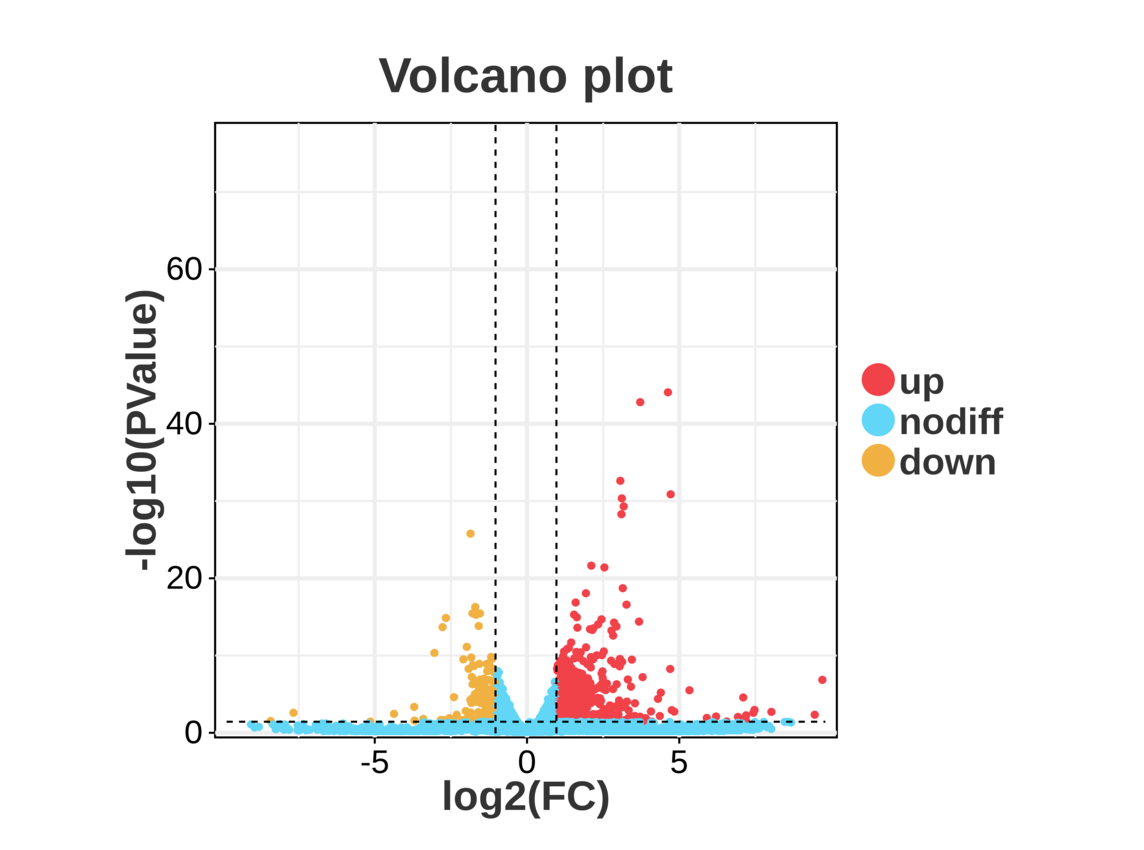

Supplement: Supplementary file 2 [file mmc2.docx]
